# Long-term use of interferon-β in multiple sclerosis increases Vδ1^−^Vδ2^−^Vγ9^−^ γδ T cells that are associated with a better outcome

**DOI:** 10.1186/s12974-019-1574-5

**Published:** 2019-09-13

**Authors:** Guzailiayi Maimaitijiang, Mitsuru Watanabe, Koji Shinoda, Noriko Isobe, Yuri Nakamura, Katsuhisa Masaki, Takuya Matsushita, Yasunobu Yoshikai, Jun-ichi Kira

**Affiliations:** 1 Department of Neurology, Neurological Institute, Graduate School of Medical Sciences, Kyushu University, 3-1-1 Maidashi, Higashi-ku, Fukuoka, 812-8582 Japan; 2 Department of Neurological Therapeutics, Neurological Institute, Graduate School of Medical Sciences, Kyushu University, 3-1-1 Maidashi, Higashi-ku, Fukuoka, 812-8582 Japan; 3 Division of Host Defense, Medical Institute of Bioregulation, Kyushu University, 3-1-1 Maidashi, Higashi-ku, Fukuoka, 812-8582 Japan

**Keywords:** Multiple sclerosis, Disease-modifying therapy, Interferon-β, γδ T cell

## Abstract

**Background:**

We previously reported that Vδ2^+^Vγ9^+^ γδ T cells were significantly decreased in multiple sclerosis (MS) patients without disease-modifying therapies (untreated MS) and were negatively correlated with Expanded Disability Status Scale (EDSS) scores, suggesting protective roles of Vδ2^+^Vγ9^+^ γδ T cells. Interferon-β (IFN-β) is one of the first-line disease-modifying drugs for MS. However, no previous studies have reported changes in γδ T cell subsets under IFN-β treatment. Therefore, we aimed to clarify the effects of the long-term usage of IFN-β on γδ T cell subsets in MS patients.

**Methods:**

Comprehensive flow cytometric immunophenotyping was performed in 35 untreated MS and 21 MS patients on IFN-β for more than 2 years (IFN-β-treated MS) including eight super-responders fulfilling no evidence of disease activity criteria, and 44 healthy controls (HCs).

**Results:**

The percentages of Vδ2^+^Vγ9^+^ cells in γδ T cells were significantly lower in untreated and IFN-β-treated MS patients than in HCs. By contrast, the percentages of Vδ1^−^Vδ2^−^Vγ9^−^ cells in γδ T cells were markedly higher in IFN-β-treated MS patients than in HCs and untreated MS patients (both *p* < 0.001). A significant negative correlation between the percentages of Vδ2^+^Vγ9^+^ cells in γδ T cells and EDSS scores was confirmed in untreated MS but not evident in IFN-β-treated MS. Moreover, class-switched memory B cells were decreased in IFN-β-treated MS compared with HCs (*p* < 0.001) and untreated MS patients (*p* = 0.006). Interestingly, the percentages of Vδ1^−^Vδ2^−^Vγ9^−^ cells in γδ T cells were negatively correlated with class-switched memory B cell percentages in all MS patients (*r* = − 0.369, *p* = 0.005), and the percentages of Vδ1^−^Vδ2^−^Vγ9^−^ cells in Vδ1^−^Vδ2^−^ γδ T cells were negatively correlated with EDSS scores only in IFN-β super-responders (*r* = − 0.976, *p* < 0.001).

**Conclusions:**

The present study suggests that long-term usage of IFN-β increases Vδ1^−^Vδ2^−^Vγ9^−^ γδ T cells, which are associated with a better outcome, especially in IFN-β super-responders. Thus, increased Vδ1^−^Vδ2^−^Vγ9^−^ cells together with decreased class-switched memory B cells may contribute to the suppression of disease activity in MS patients under IFN-β treatment.

**Electronic supplementary material:**

The online version of this article (10.1186/s12974-019-1574-5) contains supplementary material, which is available to authorized users.

## Background

Multiple sclerosis (MS) is an inflammatory demyelinating disease of the central nervous system (CNS) mediated by autoreactive T cells [1]. T cells can be divided into αβ T cells and γδ T cells according to the type of T cell receptor (TCR) they express: αβ T cells express TCR α and β chains while γδ T cells express TCR γ and δ chains. To date, αβ T cells such as interferon (IFN)-γ-secreting helper T (Th) 1 cells, interleukin (IL)-17-secreting Th17 cells, and regulatory T (Treg) cells have attracted much attention in elucidating the mechanisms of MS.

In addition, γδ T cells are also assumed to be involved in the pathogenesis of various inflammatory diseases [2], although γδ T cells comprise < 5% of lymphocytes in the peripheral blood [3]. In MS, γδ T cells are increased in the blood and cerebrospinal fluid [4], and are present in chronic active brain lesions [5–8]. We previously reported that deletion-type copy number variation (CNV) at TCRα and γ loci greatly enhanced susceptibility to MS [9]. A deletion-type CNV at the TCRα locus also covers TCRδ genes [3]. Based on these facts, we hypothesized that a deviation in TCRγδ gene rearrangement contributes to the pathogenesis of MS. Recently, we conducted comprehensive flow cytometric immunophenotyping in MS patients without disease-modifying therapies (DMTs) (defined as untreated MS), and reported that percentages of Vδ2^+^ and Vδ2^+^Vγ9^+^ cells in γδ T cells were decreased and that the Vδ1/Vδ2 ratio was increased compared with healthy controls (HCs) [10]. In this study, Vδ2^+^Vγ9^+^ γδ T cells showed a negative correlation with disability and a positive correlation with the percentages of Treg in CD4^+^ T cells, suggesting the protective roles of Vδ2^+^Vγ9^+^ γδ T cells.

IFN-β is a widely used first-line DMT for MS. IFN-β has pleiotropic effects on the peripheral immune system including the reduction of pathogenic Th1 and Th17 cells and an increase in IL-10-producing Treg cells, which are thought to be beneficial for MS [11]. In addition, IFN-β treatment reduced CD27^+^ memory B cells, which are thought to drive MS, whereas transitional B cells producing IL-10 and exerting regulatory functions were increased [12–14]. However, no previous studies have reported changes in γδ T cell subsets under IFN-β treatment. Therefore, the present study investigated the phenotypic changes of γδ T cell subsets by IFN-β treatment, which is associated with therapeutic effects in MS.

## Methods

### Study subjects

We enrolled 35 untreated MS and 21 IFN-β-treated MS patients and 44 HCs. All patients were thoroughly examined and regularly followed up at Kyushu University Hospital, Fukuoka, Japan. Diagnoses of MS and MS subtype were based on the prevailing criteria at the commencement of the study [15]; however, all enrolled patients also fulfilled the newly published revised criteria [16]. All MS patients were in the remission phase and negative for anti-aquaporin 4 antibody. Disease severity was evaluated by the Kurtzke Expanded Disability Status Scale (EDSS) [17] and MS Severity Score (MSSS) [18]. Untreated MS patients were not under any DMTs or corticosteroids for at least 6 months prior to the immunophenotyping. IFN-β-treated MS patients received either IFN-β-1a (11 patients) or IFN-β-1b (ten patients) for more than 2 years at the time of sampling (range, 3 to 20 years; median 7.0 [interquartile range (IQR) = 5.0–10.0] years). They were then separated into two groups, a ‘no-evidence of disease activity’ (NEDA) group (*n* = 8) representing super-responders, which was defined by no relapses, no EDSS progression, and no MRI activity (no new/enlarging T2 lesions or no gadolinium-enhancing lesions) [19] at least over the preceding 2 years [20, 21], and an ‘evidence of disease activity’ (EDA) group (*n* = 13) representing partial responders, who did not fulfill the definition of NEDA based on clinical and radiographic evaluation. Untreated MS patients were also classified into NEDA and EDA groups (*n* = 19 and 13, respectively) based on their disease activity over the preceding 2 years (three patients were excluded because of insufficient clinical information of disease activity over the preceding 2 years). All patients were enrolled between March 1, 2016 and May 28, 2017. The present study was approved by the Ethical Committee of Kyushu University and conducted with written informed consent from all participants according to the World Medical Association Declaration of Helsinki.

The clinical features of all participants are summarized in Table 1. The proportion of females and age at examination did not significantly differ between untreated and IFN-β-treated MS patients, while participants in both groups of MS patients were significantly older than HCs (both *p* < 0.001). Untreated MS patients had a higher proportion of females compared with HCs (*p* = 0.023). Disease duration, EDSS scores, and MSSS at examination were comparable between untreated and IFN-β-treated MS groups. Comparisons of IFN-β-treated MS patients in the NEDA and EDA groups showed that disease duration at examination, disease duration at IFN-β initiation, duration of IFN-β treatment, EDSS scores, and MSSS at IFN-β initiation were not significantly different. Changes in EDSS and MSSS when measured at IFN-β initiation and examination (ΔEDSS and ΔMSSS, respectively) were lower in the NEDA group than in the EDA group as expected (*p* = 0.003 and 0.013, respectively, Additional file 1: Table S1), and EDSS scores and MSSS at examination tended to be lower in the NEDA group than in the EDA group (*p* = 0.062 and 0.060, respectively). Moreover, age at examination in both NEDA and EDA groups was older than in HCs (*p* = 0.004 and 0.010, respectively). The clinical features of untreated MS patients in the NEDA and EDA groups were not significantly different, except for ΔEDSS and ΔMSSS, which were significantly lower in the NEDA group compared with the EDA group (*p* < 0.001, both) (Additional file 1: Table S2). A comparison between relapsing-remitting MS (RRMS) vs. progressive MS (PMS) including primary progressive MS (PPMS) and secondary progressive MS (SPMS) subtypes showed that EDSS scores and MSSS at examination were significantly higher in PMS than RRMS for both untreated and IFN-β-treated MS patients while age at examination was significantly higher in PMS than RRMS for untreated MS patients (*p* = 0.043) (Additional file 1: Table S3).

**Table 1:** Clinical demographics of multiple sclerosis patients and healthy controls

	Untreated MS (*n* = 35)	MS w/ IFN-β (*n* = 21)	HCs (*n* = 44)	*p* value		
				Untreated MS vs. HCs	MS w/ IFN-β vs. HCs	Untreated MS vs. MS w/ IFN-β
Female, *n* (%)	30 (85.7)	15 (71.4)	27 (61.4)	0.023	NS	NS
Age at examination, years	50.0 (37.0–59.0)	45.0 (42.5–48.5)	35.0 (32.3–43.8)	< 0.001	< 0.001	NS
Age at disease onset, years	31.0 (24.0–37.0)	30.0 (24.0–34.0)	–	–	–	NS
Disease duration at examination, years	13.6 (7.80–20.9)	15.4 (10.7–20.8)	–	–	–	NS
Subtype (RRMS / SPMS / PPMS), *n* (%)	26/6/3 (74.3/17.1/8.6)	13/7/1 (61.9/33.3/4.8)	–	–	–	NS
EDSS score at examination	2.0 (1.0–4.5)	3.0 (1.75–6.0)	–	–	–	NS
MSSS at examination	2.44 (0.38–6.46)	2.93 (1.00–7.15)	–	–	–	NS
Disease duration at IFN-β initiation, years	–	6.58 (1.92–12.1)	–	–	–	–
EDSS score at IFN-β initiation	–	2.5 (1.75–5.25)	–	–	–	–
MSSS at IFN-β initiation	–	5.24 (2.38–7.56)	–	–	–	–
Years of IFN-β treatment	–	7.0 (5.0–10.0)	–	–	–	–

Values are the median (IQR) or count (%)

*EDSS* Expanded Disability Status Scale, *HCs* healthy controls, *IFN*-*β* interferon-β, *IQR* interquartile ranges, *MS* multiple sclerosis, *MSSS* MS severity score, *NS* not significant, *PPMS* primary progressive MS, *RRMS* relapsing-remitting MS, *SPMS* secondary progressive MS, *w*/ with

### Antibodies and flow cytometric analysis

Sample preparation and detailed procedures were described previously [10]. In brief, peripheral blood mononuclear cells were collected by density gradient centrifugation using Lymphoprep tubes (Axis-shield Poc AS, Oslo, Norway) containing Ficoll-Paque (GE Healthcare, Little Chalfont, UK) and then suspended in RPMI-1640 medium (Wako, Osaka, Japan) supplemented with 10% fetal bovine serum. For intracellular staining, cell suspensions were incubated with 25 ng/ml of phorbol 12-myristate 13-acetate and 1 μg/ml of ionomycin in the presence of 10 μg/ml of Brefeldin A (all purchased from Sigma-Aldrich, St. Louis, MO, USA) for 4 h at 37 °C. The following fluorochrome-conjugated anti-human monoclonal antibodies were used in this study: anti-CD3 (clone: SK-7), anti-CD8 (RPA-T8), anti-CD14 (M5E2), anti-CD19 (HIB-19), anti-CD20 (2H7), anti-CD27 (M-T271), anti-CD45RA (HI100), anti-CD127 (HIlL-7R-M21), anti-CCR7 (150503), anti-HLR-DR (G-46-6), anti-IgD (IA6-2), anti-IL17A (N49-653), anti-TCR Vδ2 (B6), anti-TCR Vγ9 (B3), TCR γδ (B1) (BD Biosciences, Franklin Lakes, NJ, USA), and anti-CD4 (RPA-T4), anti-CD24 (MIL-5), anti-CD25 (BC96), anti-CD38 (HB-7), anti-CD3 (UCHT1), anti-IFN-γ (4S.B3), anti-IL-4 (MP4-25D2), anti-granulocyte macrophage colony-stimulating factor (GM-CSF) (BVD-21C11) (BioLegend, San Diego, CA, USA), anti-TCR Vδ1 (TS-1) (Thermo Fisher Science, Waltham, MA, USA), anti-IL-17A (eBio64DEC17), and anti-TCR αβ (IP-26) (eBioscience, San Diego, CA, USA) antibodies. Stained cells were analyzed on a FACSVerse flow cytometer (BD Biosciences). The data were analyzed using FlowJo software (Tree Star, Ashland, OR, USA). The immunophenotyping strategy for γδ T cell subsets is shown in Additional file 2: Figure S1a.

### Statistical analysis

Categorical variables were described by counts and percentages, and continuous and ordinal variables by median and IQRs. Demographic features of participants were compared using Fisher’s exact test or the Wilcoxon test. When comparing the proportions of MS subtype between groups, a likelihood ratio chi-square test was used because it includes more than two categories. The Wilcoxon test was used to compare the percentages of each cell subtype among the two groups (RRMS vs. PMS). The Kruskal-Wallis non-parametric test was used to compare percentages among three groups (untreated MS vs. IFN-β-treated MS vs. HCs, or NEDA vs. EDA vs. HCs). If there was statistical significance by the Kruskal-Wallis analysis, multivariate linear regression analyses adjusting for age and sex were performed for comparison between two groups, and adjusted *p* values (*p*^*adj*^) are shown. Correlations among continuous scales were calculated using Spearman’s rank correlation coefficient. Statistical analysis was performed using JMP® Pro version 14.0.0 software (SAS Institute, Cary, NC, USA). The significance level was set at *p* < 0.05.

## Results

### γδ T cell subsets

First, we assessed differences in the percentages of Vδ1^+^, Vδ2^+^, and Vδ1^−^Vδ2^−^ cells in γδ T cells between untreated MS, IFN-β-treated MS, and HCs. Vδ2^+^ and Vδ1^−^Vδ2^−^ cells were significantly different between groups (both *p* < 0.001, Table 2, Additional file 2: Figure S1b). The percentage of Vδ2^+^ γδ T cells was significantly lower in untreated MS patients and IFN-β-treated MS patients compared with that in HCs (*p*^*adj*^ = 0.047 and *p*^*adj*^ < 0.001, respectively). Vδ2^+^Vγ9^+^ γδ T cells were also lower in both MS patient groups than in HCs (*p*^*adj*^ = 0.043 and *p*^*adj*^ < 0.001, respectively). Additionally, the percentages of Vδ2^+^ and Vδ2^+^Vγ9^+^ γδ T cells were significantly lower in IFN-β-treated MS than in untreated MS (*p*^*adj*^ = 0.017 and 0.016, respectively). More interestingly, the percentage of Vδ1^−^Vδ2^−^ γδ T cells was significantly higher in IFN-β-treated MS patients than in untreated MS and HCs (*p*^*adj*^ = 0.002 and *p*^*adj*^ < 0.001, respectively). Moreover, the percentage of Vδ1^−^Vδ2^−^Vγ9^−^ γδT cells in IFN-β-treated MS patients was higher than in untreated MS patients and HCs (both *p*^*adj*^ < 0.001). The percentage of Vδ1^−^Vδ2^−^Vγ9^+^ cells in IFN-β-treated MS patients was lower than in HCs (*p*^*adj*^ = 0.009). These results suggest that IFN-β increased the percentage of Vδ1^−^Vδ2^−^ γδ T cells, especially Vδ1^−^Vδ2^−^Vγ9^−^ γδ T cells, and decreased the percentage of Vδ2^+^ γδ T cells, especially Vδ2^+^Vγ9^+^ γδ T cells.

**Table 2:** Comparison of the percentages of γδ T cell subsets between untreated MS patients, IFN-β-treated MS patients, and healthy controls

	Untreated MS (*n* = 35)	MS w/ IFN-β (*n* = 21)	HCs (*n* = 44)	*p* value (K-W test)	*p*^*adj*^ value		
					Untreated MS vs. HCs	MS w/ IFN-β vs. HCs	Untreated MS vs. MS w/ IFN-β
Vδ1^+^	29.0 (14.0–53.9)	29.6 (13.7–48.3)	18.4 (11.6–34.2)	NS	–	–	–
Vδ1^+^Vγ9^+^	3.91 (1.26–10.2)	3.00 (1.60–5.17)	2.15 (1.12–4.71)	NS	–	–	–
Vδ1^+^Vγ9^–^	24.3 (10.2–40.8)	23.5 (10.0–38.3)	15.5 (8.47–31.5)	NS	–	–	–
Vδ2^+^	32.9 (13.3–52.3)	21.2 (5.07–33.4)	54.9 (31.7–65.9)	< 0.001	0.047	< 0.001	0.017
Vδ2^+^Vγ9^+^	32.7 (12.9–52.2)	21.1 (4.70–32.1)	54.3 (31.1–65.7)	< 0.001	0.043	< 0.001	0.016
Vδ2^+^Vγ9^–^	0.16 (0.07–0.46)	0.16 (0.00–0.44)	0.08 (0.03–0.29)	NS	–	–	–
Vδ1^−^Vδ2^−^	22.8 (15.5–39.5)	35.6 (27.1–58.2)	22.8 (17.1–30.3)	0.001	NS	< 0.001	0.002
Vδ1^−^Vδ2^−^Vγ9^+^	1.23 (0.36–2.91)	1.10 (0.33–2.53)	2.42 (1.25–3.83)	0.010	NS	0.009	NS
Vδ1^−^Vδ2^−^Vγ9^−^	22.0 (14.7–36.1)	33.1 (24.5–55.3)	18.1 (10.7–26.4)	< 0.001	NS (0.073)	< 0.001	< 0.001

Values are the median (IQR). Percentages of each population in total γδ T cells are shown

*p* values (K-W test) were obtained by Kruskal-Wallis analyses, and if they were statistically significant, then *p*^*adj*^ values were calculated using multivariate linear regression analyses adjusted for age and sex

*HCs* healthy controls, *IFN*-*β* interferon-β, *IQR* interquartile ranges, *K*-*W* Kruskal-Wallis, *MS* multiple sclerosis, *NS* not significant, *w*/ with

### Cytokine production by γδ T cell subsets

Next, we compared the effect of IFN-β treatment on the cytokine production of γδ T cells. In Vδ1^+^, Vδ2^+^, and Vδ1^−^Vδ2^−^ γδ T cells, the percentages of IFN-γ-producing cells were lower in both MS groups compared with HCs (Table 3), and there were no significant differences between untreated MS and IFN-β-treated MS groups.

**Table 3:** Comparison of the percentages of cytokine-producing γδ T cell subsets between untreated MS patients, IFN-β-treated MS patients, and healthy controls

	Untreated MS (*n* = 34)	MS w/ IFN-β (*n* = 21)	HCs (*n* = 44)	*p* value (K-W test)	*p*^*adj*^ value		
					Untreated MS vs. HCs	MS w/ IFN-β vs. HCs	Untreated MS vs. MS w/ IFN-β
In Vδ1^+^ γδ T cells							
IL-17A^+^	0.13 (0.00–0.49)	0.40 (0.02–1.03)	0.18 (0.01–0.72)	NS	–	–	–
IFN-γ^+^	36.0 (17.3–46.2)	29.6 (7.35–39.3)	45.8 (24.4–60.8)	0.012	0.017	0.004	NS
IL-17A^+^IFN-γ^+^	0.03 (0.00–0.13)	0.17 (0.00–0.55)	0.08 (0.00–0.31)	NS	–	–	–
IL-17A^−^IFN-γ^−^	64.0 (53.8–81.8)	69.3 (60.7–91.9)	54.1 (39.1–75.5)	0.011	0.016	0.004	NS
In Vδ2^+^ γδ T cells							
IL-17A^+^	0.07 (0.00–0.42)	0.00 (0.00–1.63)	0.13 (0.01–0.52)	NS	–	–	–
IFN-γ^+^	43.1 (9.70–81.2)	39.4 (23.7–64.4)	83.6 (69.1–92.7)	< 0.001	< 0.001	< 0.001	NS
IL-17A^+^IFN-γ^+^	0.00 (0.00–0.12)	0.00 (0.00–0.50)	0.11 (0.01–0.42)	0.011	NS	NS	NS
IL-17A^−^IFN-γ^−^	56.0 (18.7–90.2)	60.6 (35.7–76.4)	16.0 (7.30–30.9)	< 0.001	< 0.001	< 0.001	NS
In Vδ1^−^Vδ2^−^ γδ T cells							
IL-17A^+^	0.58 (0.26–1.40)	0.79 (0.40–1.87)	1.24 (0.66–2.81)	0.030	NS	NS	NS
IFN-γ^+^	29.1 (11.2–40.1)	18.3 (11.6–34.4)	47.1 (32.5–58.2)	< 0.001	< 0.001	< 0.001	NS
IL-17A^+^IFN-γ^+^	0.23 (0.01–0.51)	0.41 (0.08–0.65)	0.35 (0.19–1.22)	NS	–	–	–
IL-17A^−^IFN-γ^−^	70.7 (59.4–88.4)	81.4 (64.7–87.8)	51.7 (41.3–66.9)	< 0.001	< 0.001	< 0.001	NS

Values are the median (IQR). Percentages of each population are shown. Data from one untreated MS patients was missing

*p* values (K-W test) were obtained by Kruskal-Wallis analyses, and if they were statistically significant, then *p*^*adj*^ values were calculated using multivariate linear regression analyses adjusted for age and sex

*HCs* healthy controls, *IFN* interferon, *IL* interleukin, *IQR* interquartile ranges, *K*-*W* Kruskal-Wallis, *MS* multiple sclerosis, *NS* not significant, *w*/ with

### αβ T cell subsets

Next, the percentages of αβ T cell subsets between MS patients and HCs were compared. In CD4^+^ T cells, percentages of Treg cells were different among the three groups, but after adjusting for age and sex, the differences were not significant. There were no significant differences in other CD4^+^ T cell subsets (Additional file 1: Table S4). In CD8^+^ T cells, the percentages of naïve T cells and central memory T cells were higher in IFN-β-treated MS patients than in untreated MS patients (*p*^*adj*^ = 0.031 and 0.013, respectively, Additional file 1: Table S5). When analyzing cytokine production in αβ T cells, percentages of IL-17A^+^, IL-4^+^, GM-CSF^+^, and IL-17A^+^GM-CSF^+^ CD4^+^ T cells were lower in untreated MS patients than in HCs (*p*^*adj*^ = 0.017, 0.011, 0.002, and 0.010, respectively), and percentages of GM-CSF^+^ CD4^+^ T cells were also lower in IFN-β-treated MS than in HCs (*p*^*adj*^ = 0.034, Additional file 1: Table S6). However, there were no significant differences in cytokine producing CD4^+^ T cells between the two MS groups. The percentage of IL-17A^+^IFN-γ^+^ CD4^+^ T cells was significantly different among the three groups, but it was not significant after adjusting for age and sex. For CD8^+^ T cells, percentages of IL-17A^+^, IFN-γ^+^, and IL-17A^+^IFN-γ^+^ cells were not different among the three groups.

### B cell subsets

The percentage of circulating memory B cells was significantly different between groups (*p* < 0.001, Table 4). The percentages of memory B cells and class-switched memory B cells were specifically decreased in IFN-β-treated MS, but not in untreated MS patients (for memory B cells: IFN-β-treated MS vs. untreated MS, *p*^*adj*^ = 0.009; IFN-β-treated MS vs. HCs, *p*^*adj*^ < 0.001; untreated MS vs. HCs, *p*^*adj*^ = 0.669; for class-switched memory B cells: IFN-β-treated MS vs. untreated MS, *p*^*adj*^ = 0.006; IFN-β-treated MS vs. HCs, *p*^*adj*^ < 0.001; untreated MS vs. HCs, *p*^*adj*^ = 0.984). However, percentages of non-class-switched memory B cells were lower in IFN-β-treated MS and tended to be lower in untreated MS than in HCs (*p*^*adj*^ < 0.001 and *p*^*adj*^ = 0.058, respectively) and they were not different between IFN-β-treated MS and untreated MS. There were no significant differences in the percentages of naïve B cells, transitional B cells, and plasmablasts between MS patients and HCs. Our results suggest that IFN-β therapy specifically reduces the percentage of memory B cells, especially class-switched memory B cells (Table 4).

**Table 4:** Comparison of the percentages of B cell subsets between untreated MS patients, IFN-β-treated MS patients, and healthy controls

	Untreated MS (*n* = 35)	MS w/ IFN-β (*n* = 21)	HCs (*n* = 44)	*p* value (K-W test)	*p*^*adj*^ value		
					Untreated MS vs. HCs	MS w/ IFN-β vs. HCs	Untreated MS vs. MS w/ IFN-β
Naïve (CD27^−^IgD^+^)	46.3 (27.9–56.8)	48.9 (37.7–58.9)	51.8 (41.4–60.6)	NS	–	–	–
Memory (CD27^+^)	14.8 (10.3–27.0)	9.84 (6.89–15.2)	22.7 (16.9–29.9)	< 0.001	NS	< 0.001	0.009
CS^+^ memory (CD27^+^IgD^−^)	12.4 (9.27–24.8)	7.97 (6.10–14.0)	18.8 (14.4–25.2)	< 0.001	NS	< 0.001	0.006
CS^−^ memory (CD27^+^IgD^+^)	1.91 (0.83–2.68)	0.99 (0.85–1.67)	3.49 (2.49–4.67)	< 0.001	NS (0.058)	< 0.001	NS
Plasmablasts (CD38^high^CD20^−^)	0.31 (0.14–0.65)	0.32 (0.25–0.53)	0.37 (0.24–0.67)	NS	–	–	–
Transitional (CD24^high^CD38^high^)	3.16 (1.75–5.93)	5.90 (1.92–7.66)	3.14 (2.32–4.45)	NS	–	–	–

Values are the median (IQR). Percentages of each population in total B cells are shown

*p* values (K-W test) were obtained by Kruskal-Wallis analyses, and if they were statistically significant, then *p*^*adj*^ values were calculated using multivariate linear regression analyses adjusted for age and sex

*CS*^+^ class-switched, *CS*^−^ non-class-switched, *HCs* healthy controls, *IFN*-*β* interferon-β, *IQR* interquartile ranges, *K*-*W* Kruskal-Wallis, *MS* multiple sclerosis, *NS* not significant, *w*/ with

### Comparisons of the percentages of γδ T cells subsets in untreated MS patients stratified to the NEDA or EDA groups

Next, we compared the percentages of γδ T cell subsets between the NEDA and EDA groups in IFN-β-treated MS. The percentages of Vδ2^+^ and Vδ2^+^Vγ9^+^ γδ T cells were similar in the NEDA and EDA groups but both were lower than in HCs (Vδ2^+^: NEDA vs. HCs, *p*^*adj*^ < 0.001; EDA vs. HCs, *p*^*adj*^ = 0.006; Vδ2^+^Vγ9^+^: NEDA vs. HCs, *p*^*adj*^ < 0.001; EDA vs. HCs, *p*^*adj*^ = 0.005, Table 5). The percentages of Vδ1^−^Vδ2^−^ and Vδ1^−^Vδ2^−^Vγ9^−^ γδ T cells in γδ T cells were similar in the NEDA and EDA groups but both were higher than in HCs (Vδ1^−^Vδ2^−^: NEDA vs. HCs, *p*^*adj*^ = 0.033; EDA vs. HCs, *p*^*adj*^ < 0.001; Vδ1^−^Vδ2^−^Vγ9^−^: NEDA vs. HCs, *p*^*adj*^ = 0.011; EDA vs. HCs, *p*^*adj*^ < 0.001). The percentages of Vδ1^−^Vδ2^−^Vγ9^+^ γδ T cells in γδ T cells were lower in the EDA group and tended to be lower in the NEDA group than in HCs (*p*^*adj*^ = 0.022 and *p*^*adj*^ = 0.061, respectively), but they were not significantly different between NEDA and EDA groups. Regarding cytokine production, the percentages of IFN-γ-producing cells in Vδ2^+^ γδ T cells were lower in both IFN-β-treated groups than in HCs (NEDA vs. HCs, *p*^*adj*^ = 0.003; EDA vs. HCs, *p*^*adj*^ < 0.001) and those in Vδ1^+^ and Vδ1^−^Vδ2^−^ γδ T cells were lower only in the EDA group compared with HCs (*p*^*adj*^ = 0.001 and *p*^*adj*^ < 0.001, respectively). Moreover, IFN-γ-producing Vδ1^−^Vδ2^−^ γδ T cells were significantly lower in the EDA group than in the NEDA group (*p*^*adj*^ = 0.033, Additional file 1: Table S7).

**Table 5:** Comparison of the percentages of γδ T cell subsets in IFN-β-treated MS patients stratified to the NEDA or EDA groups

	MS w/IFN-β		HCs (*n* = 44)	*p* value (K-W test)	*p*^*adj*^ value		
	NEDA (*n* = 8)	EDA (*n* = 13)			NEDA vs. HCs	EDA vs. HCs	NEDA vs. EDA
Vδ1^+^	39.5 (21.1–55.9)	17.4 (11.6–38.5)	18.4 (11.6–34.2)	NS	–	–	–
Vδ1^+^Vγ9^+^	3.01 (1.32–10.7)	2.99 (1.60–3.94)	2.15 (1.12–4.71)	NS	–	–	–
Vδ1^+^Vγ9^–^	36.4 (17.5–51.9)	15.2 (9.18–31.9)	15.5 (8.47–31.5)	NS	–	–	–
Vδ2^+^	14.2 (3.17–25.2)	24.7 (6.33–42.9)	54.9 (31.7–65.9)	< 0.001	< 0.001	0.006	NS
Vδ2^+^Vγ9^+^	14.1 (2.76–25.0)	24.3 (6.09–42.8)	54.3 (31.1–65.7)	< 0.001	< 0.001	0.005	NS
Vδ2^+^Vγ9^–^	0.12 (0.00–0.26)	0.18 (0.06–0.84)	0.08 (0.03–0.29)	NS	–	–	–
Vδ1^−^Vδ2^−^	32.3 (20.0–61.8)	36.1 (28.4–54.0)	22.8 (17.1–30.3)	< 0.001	0.033	< 0.001	NS
Vδ1^−^Vδ2^−^Vγ9^+^	1.68 (0.30–2.83)	1.01 (0.26–2.23)	2.42 (1.25–3.83)	0.025	NS (0.061)	0.022	NS
Vδ1^−^Vδ2^−^Vγ9^–^	28.2 (17.1–60.7)	35.1 (27.5–51.0)	18.1 (10.7–26.4)	< 0.001	0.011	< 0.001	NS

Values are the median (IQR). Percentages of each population in total γδ T cells are shown

*p* values (K-W test) were obtained by Kruskal-Wallis analyses, and if they were statistically significant, then *p*^*adj*^ values were calculated using multivariate linear regression analyses adjusted for age and sex

*EDA* evidence of disease activity, *HCs* healthy controls, *IFN*-*β* interferon-β, *IQR* interquartile ranges, *K*-*W* Kruskal-Wallis, *MS* multiple sclerosis, *NEDA* no-evidence of disease activity, *NS* not significant, *w*/ with

### Differences in B cell subsets between NEDA and EDA groups in IFN-β-treated MS

Comparisons of B cell subsets between the NEDA and EDA groups in IFN-β-treated MS and HCs showed that the percentages of memory B cells, class-switched memory B cells, and non-class-switched B cells were lower in both IFN-β-treated MS groups than in HCs (memory B: NEDA vs. HCs, *p*^*adj*^ = 0.009; EDA vs. HCs, *p*^*adj*^ < 0.001; class-switched memory B: NEDA vs. HCs, *p*^*adj*^ = 0.017; EDA vs. HCs, *p*^*adj*^ < 0.001; non-class-switched B cells: NEDA vs. HCs, *p*^*adj*^ = 0.076; EDA vs. HCs, *p*^*adj*^ = 0.002, Table 6), but the percentages of these memory B cell subsets in the NEDA and EDA groups were comparable. The percentages of other B cell subsets were not different between groups.

**Table 6:** Comparison of the percentages of B cell subsets in IFN-β-treated MS patients stratified to the NEDA or EDA groups

	MS w/IFN-β		HCs (*n* = 44)	*p* value (K-W test)	*p*^*adj*^ value		
	NEDA (*n* = 8)	EDA (*n* = 13)			NEDA vs. HCs	EDA vs. HCs	NEDA vs. EDA
Naïve (CD27^−^IgD^+^)	57.9 (46.6–64.6)	47.3 (29.4–54.8)	51.8 (41.4–60.6)	NS	–	–	–
Memory (CD27^+^)	12.2 (8.75–18.3)	8.87 (6.42–14.0)	22.7 (16.9–29.9)	< 0.001	0.009	< 0.001	NS
CS^+^ Memory (CD27^+^IgD^−^)	10.8 (6.28–16.5)	7.74 (5.96–13.2)	18.8 (14.4–25.2)	< 0.001	0.017	< 0.001	NS
CS^−^ Memory (CD27^+^IgD^+^)	1.66 (0.96–3.09)	0.96 (0.70–1.25)	3.49 (2.49–4.67)	< 0.001	NS (0.076)	0.002	NS
Plasmablasts (CD38^high^CD20^−^)	0.37 (0.28–0.55)	0.32 (0.22–0.58)	0.37 (0.24–0.67)	NS	–	–	–
Transitional (CD24^high^CD38^high^)	6.73 (2.75–14.9)	4.56 (1.32–7.02)	3.14 (2.32–4.45)	NS	–	–	–

Values are the median (IQR). Percentages of each population in total B cells are shown

*p* values (K-W test) were obtained by Kruskal-Wallis analyses, and if they were statistically significant, then *p*^*adj*^ values were calculated using multivariate linear regression analyses adjusted for age and sex

*CS*^+^ class-switched, *CS*^−^ non-class-switched, *EDA* evidence of disease activity, *HCs* healthy controls, *IFN*-*β* interferon-β, *IQR* interquartile ranges, *K*-*W* Kruskal-Wallis, *MS* multiple sclerosis, *NEDA* no-evidence of disease activity, *NS* not significant, *w*/ with

### Relationship between γδ T and Treg cell subsets

**Fig. 1:**
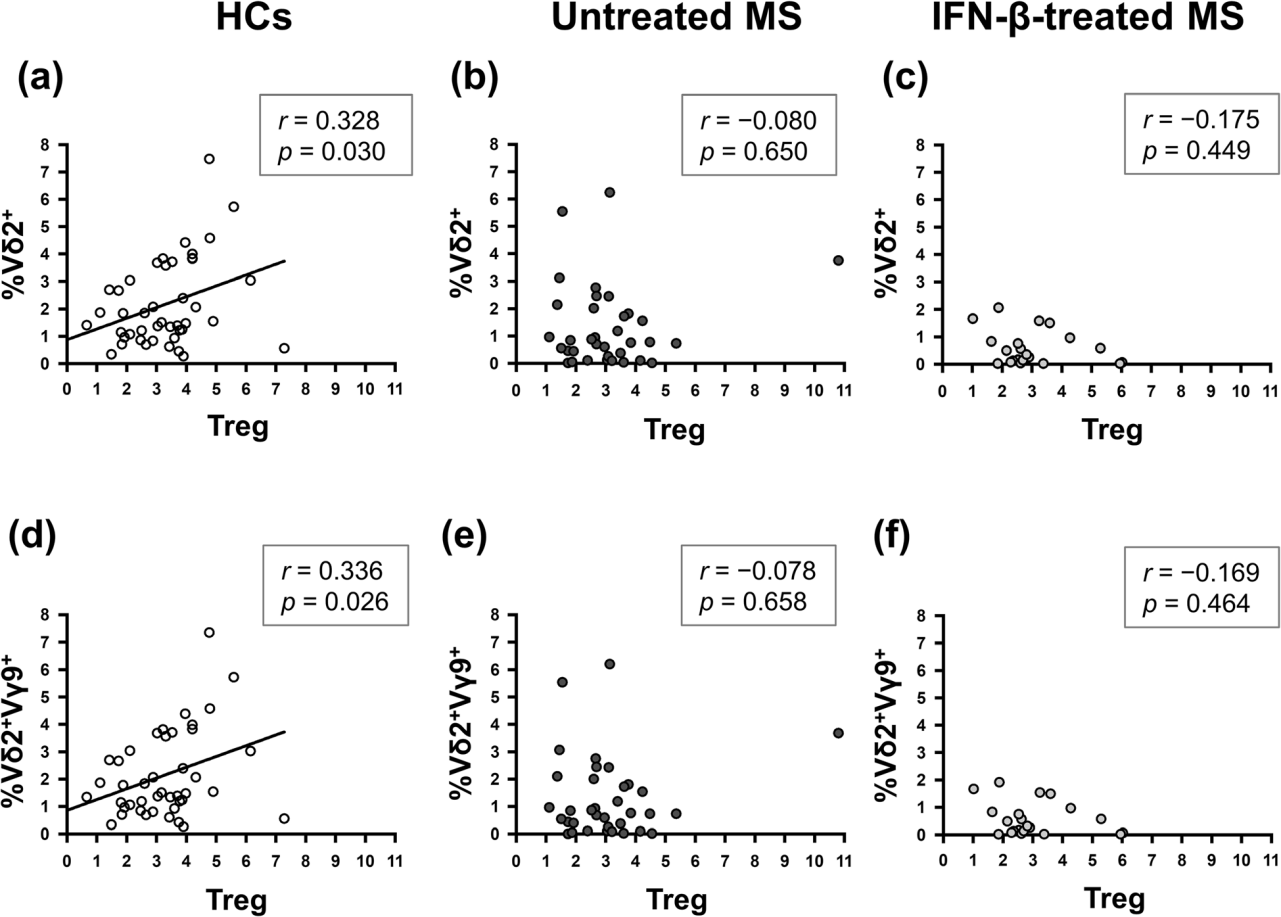
Correlation of the percentages of Vδ2^+^ and Vδ2^+^Vγ9^+^ γδ T cells with Treg cells. **a**–**c** Correlation between the percentage of Vδ2^+^ γδ T cells and Treg cells among T cells in HCs (**a**), untreated MS (**b**), and IFN-β-treated MS patients (**c**). **d**–**f** Correlation between the percentage of Vδ2^+^Vγ9^+^ γδ T cells and Treg cells among T cells in HCs (**d**), untreated MS (**e**), and IFN-β-treated MS patients (**f**). Correlations were calculated using Spearman’s rank correlation coefficient. *HC* healthy control, *IFN*-*β* interferon-β, *MS* multiple sclerosis, *Treg* regulatory T

Consistent with our previous study [10], positive correlations between the percentages of Vδ2^+^ and Treg cells in T cells, and the percentages of Vδ2^+^Vγ9^+^ γδ T cells and Treg cells in T cells were specifically found in HCs (Vδ2^+^: *r* = 0.328, *p* = 0.030; and Vδ2^+^Vγ9^+^: *r* = 0.336, *p* = 0.026, respectively) but not in untreated MS and IFN-β-treated MS (Fig. 1).

### Association between γδ T cell subsets and disease severity in MS patients

**Fig. 2:**
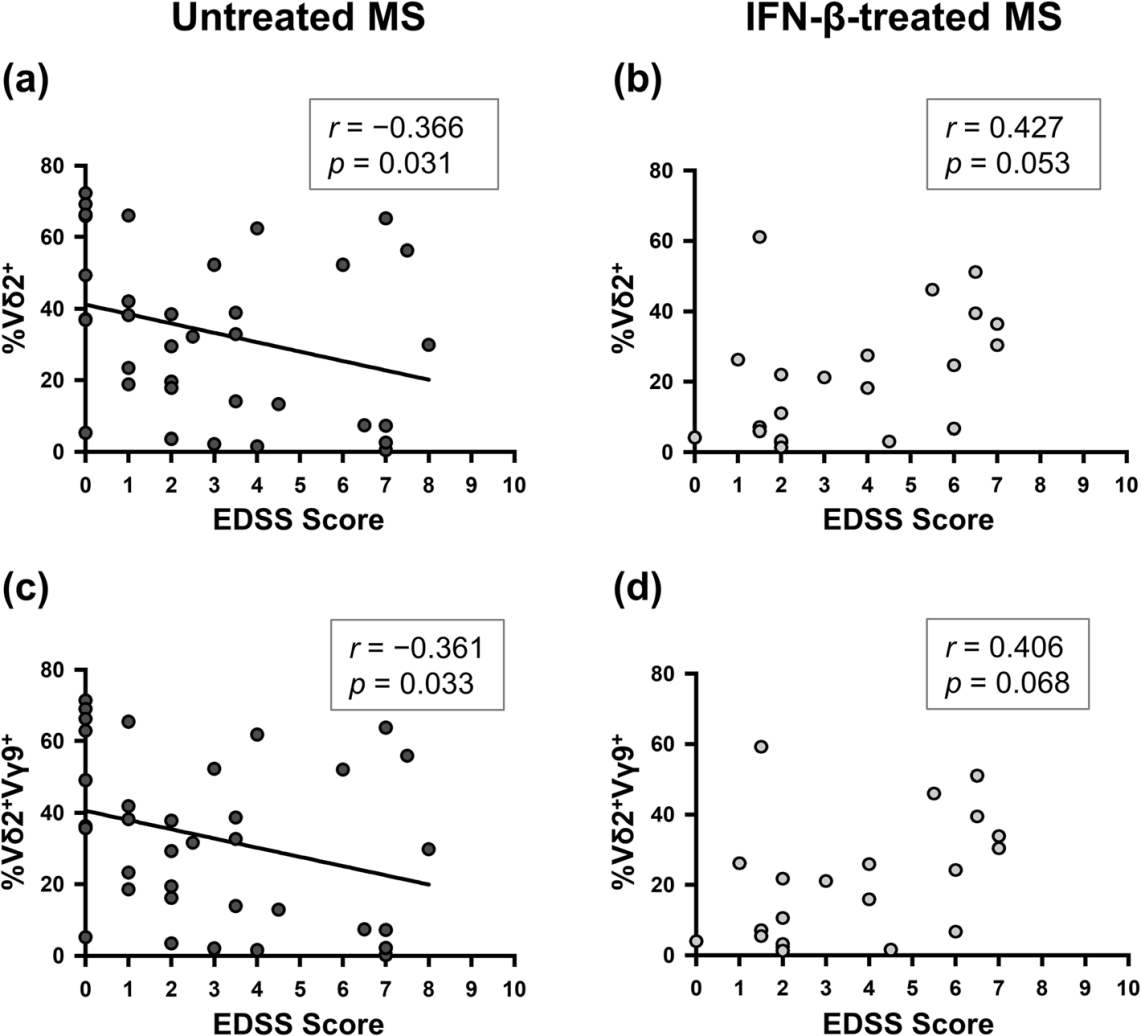
Correlation of the percentages of Vδ2^+^ and Vδ2^+^Vγ9^+^ γδ T cells with EDSS scores. **a**, **b** Correlation between the percentage of Vδ2^+^ cells in γδ T cells and EDSS scores in untreated MS (**a**) and IFN-β-treated MS patients (**b**). **c**, **d** Correlation between the percentage of Vδ2^+^Vγ9^+^ cells in γδ T cells and EDSS scores in untreated MS (**c**) and IFN-β-treated MS patients (**d**). Correlations were calculated using Spearman’s rank correlation coefficient. *EDSS* Expanded Disability Status Scale, *IFN*-*β* interferon-β, *MS* multiple sclerosis

**Fig. 3:**
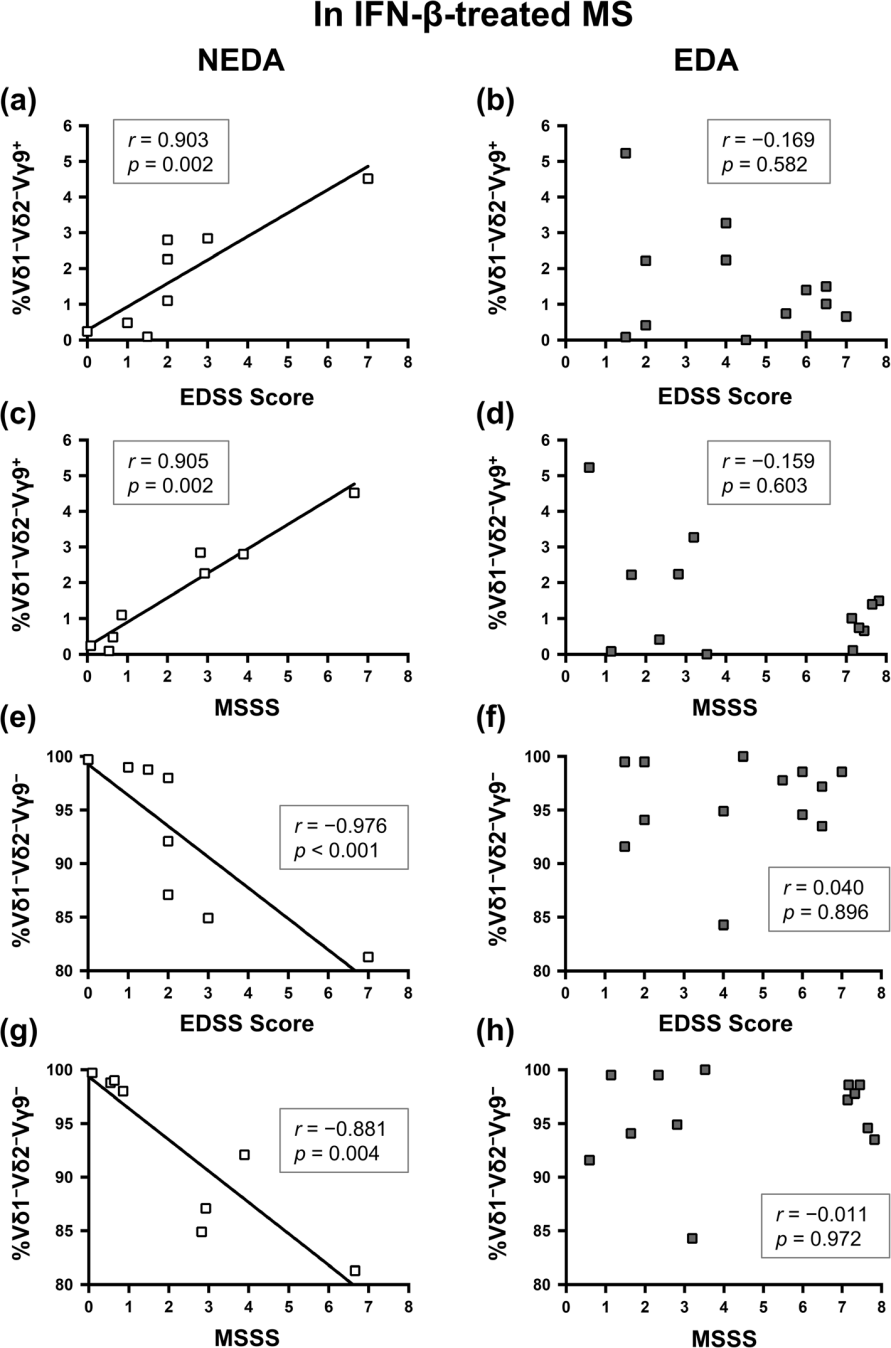
Correlations between the percentages of γδ T cell subsets and disability in IFN-β-treated MS patients. **a**–**d** Correlation between the percentage of Vδ1^−^Vδ2^−^Vγ9^+^ cells in γδ T cells and EDSS scores (**a**, **b**) or MSSS (**c**, **d**) at examination in NEDA (**a**, **c**) and EDA (**e**, **f**) groups of IFN-β-treated MS patients. **e**–**h** Correlation between the percentage of Vδ1^−^Vδ2^−^Vγ9^−^ cells in Vδ1^−^Vδ2^−^ γδ T cells and EDSS scores (**e**, **f**) or MSSS (**g**, **h**) at examination in NEDA (**e**, **g**) and EDA (**f**, **h**) groups of IFN-β-treated MS patients. Correlations were calculated using Spearman’s rank correlation coefficient. *EDA* evidence of disease activity, *EDSS* Expanded Disability Status Scale, *IFN*-*β* interferon-β, *MS* multiple sclerosis, *MSSS* multiple sclerosis severity score, *NEDA* no-evidence of disease activity

Additionally, we assessed the association between γδ T cells and disability in MS patients. As previously shown [10], the percentages of Vδ2^+^ and Vδ2^+^Vγ9^+^ cells in γδ T cells had significant negative correlations with EDSS scores in untreated MS patients (Vδ2^+^: *r* = − 0.366, *p* = 0.031; and Vδ2^+^Vγ9^+^: *r* = − 0.361, *p* = 0.033), while such correlations were lost in IFN-β-treated MS patients (Vδ2^+^: *p* = 0.053; and Vδ2^+^Vγ9^+^: *p* = 0.068, Fig. 2). Even though IFN-β-treated MS patients were divided to NEDA and EDA groups, these cells were not associated with EDSS scores and MSSS. However, the percentages of Vδ1^−^Vδ2^−^Vγ9^+^ cells in γδ T cells had a strong positive correlation with EDSS scores and MSSS only in the NEDA group (EDSS: *r* = 0.903, *p* = 0.002; and MSSS: *r* = 0.905, *p* = 0.002), but such correlations were not observed in the EDA group (Fig. 3a–d). Moreover, the proportion of Vδ1^−^Vδ2^−^Vγ9^−^ cells in Vδ1^−^Vδ2^−^ γδ T cells were negatively correlated with EDSS scores and MSSS in the NEDA group (EDSS: *r* = − 0.976, *p* < 0.001; and MSSS: *r* = 0.881, *p* = 0.004), but not the EDA group (Fig. 3e–h). These data suggest that increased Vδ1^−^Vδ2^−^Vδ9^−^ γδT cells induced by the long-term use of IFN-β are associated with a better outcome in IFN-β super-responders.

### Association between B cell subsets and disease severity in MS patients treated by IFN-β

**Fig. 4:**
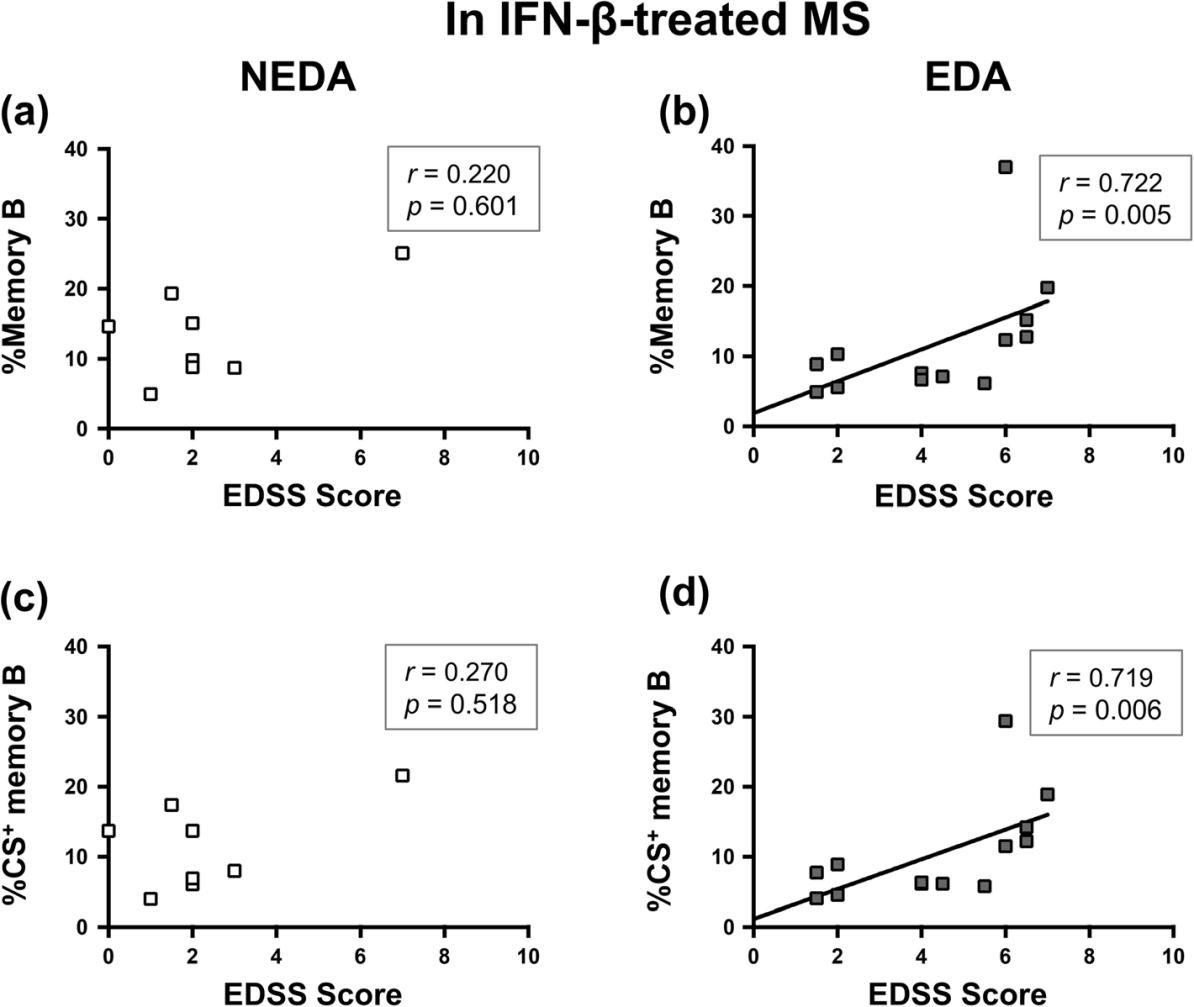
Correlations of memory B cell proportions with disability in IFN-β treated MS patients. **a**, **b** Correlation between the percentage of memory B cells and EDSS scores in the NEDA (**a**) and EDA (**b**) groups of IFN-β-treated MS patients. **c**, **d** Correlation between the percentage of class-switched memory B cells and EDSS scores in the NEDA (**c**) and EDA (**d**) groups of IFN-β-treated MS patients. Correlations were calculated using Spearman’s rank correlation coefficient. *CS*^+^ class-switched, *EDA* evidence of disease activity, *IFN*-*β* interferon-β, *MS* multiple sclerosis, *NEDA* no-evidence of disease activity

When we examined the association between B cell subsets and disease severity, the percentages of memory B cells and class-switched memory B cells were positively associated with EDSS scores only in the EDA group of IFN-β-treated MS patients (*r* = 0.722, *p* = 0.005; *r* = 0.719, *p* = 0.006, respectively), but not in the NEDA group (*p* = 0.601 and 0.518, respectively) (Fig. 4).

### Relationship between γδ T and B cell subsets

**Fig. 5:**
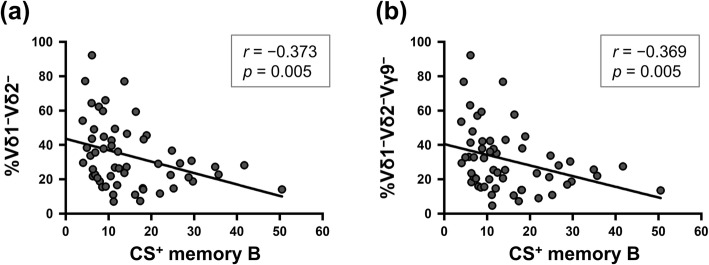
Correlations of γδ T cell subsets and memory B cell proportions in patients with MS. Negative correlation between the percentage of Vδ1^−^Vδ2^−^ cells in γδ T cells and class-switched memory B cells (**a**) and between the percentages of Vδ1^−^Vδ2^−^Vγ9^−^ cells in γδ T cells and class-switched memory B cells (**b**) in patients with MS. Data from untreated MS patients and IFN-β-treated MS patients are included. Correlations were calculated using Spearman’s rank correlation coefficient. *CS*^+^ class-switched, *IFN*-*β* interferon-β, *MS* multiple sclerosis

Interestingly, we observed the percentages of Vδ1^−^Vδ2^−^ cells and Vδ1^−^Vδ2^−^Vγ9^−^ cells in γδ T cells were negatively correlated with class-switched memory B cells when analyzing all MS patients (Vδ1^−^Vδ2^−^: *r* = − 0.373, *p* = 0.005; and Vδ1^−^Vδ2^−^Vγ9^−^: *r* = − 0.369, *p* = 0.005, Fig. 5).

### Comparison of untreated MS patients in the NEDA and EDA groups

The percentages of each γδ T cell subset were similar in untreated MS patients in the NEDA and EDA groups (Additional file 1: Table S8). The percentage of plasmablasts, but not other B cell subsets, were higher in the EDA group than in the NEDA group (*p*^*adj*^ = 0.008) (Additional file 1: Table S9). The proportion of Vδ1^−^Vδ2^−^Vγ9^−^ cells in Vδ1^−^Vδ2^−^ γδ T cells was not associated with the EDSS score nor MSSS in the NEDA and EDA groups (Additional file 2: Figure S2), although the percentage of Vδ1^−^Vδ2^−^Vγ9^+^ cells in γδ T cells had a negative correlation with EDSS score and MSSS in the NEDA group (EDSS: *r* = − 0.514, *p* = 0.024; and MSSS: *r* = − 0. 593, *p* = 0.008) but not in the EDA group. Memory B cell and class-switched memory B cell percentages were not associated with EDSS scores in the NEDA and EDA groups (Additional file 2: Figure S3).

### Differences in T and B cell subsets between RRMS and PMS

Finally, we compared the percentages of T and B cell subsets between RRMS and PMS (Additional file 1: Tables S10–S15). In untreated MS patients, the percentages of naïve T cells in CD4^+^ T cells and Vδ1^−^Vδ2^−^Vγ9^+^ cells in γδ T cells were higher and the percentage of effector memory T cells in CD4^+^ T cells was lower in RRMS than in PMS (*p* = 0.002, 0.050, and 0.004, respectively). Otherwise, no significant difference in any subsets was found between RRMS and PMS. In IFN-β-treated MS patients, no significant difference was found between RRMS and PMS, except that the percentages of naïve B cells and transitional B cells were significantly higher in RRMS than in PMS (*p* < 0.001 and *p* = 0.015, respectively). Furthermore, in RRMS, the percentages of naïve B cells and transitional B cells were higher in IFN-β-treated MS patients than in untreated MS patients (*p*^*adj*^ = 0.010 and 0.001, respectively). These findings suggest that IFN-β treatment increased the percentage of Vδ1^−^Vδ2^−^Vγ9^−^ γδ T cells irrespective of clinical subtype.

## Discussion

Our comprehensive immunophenotyping study of peripheral blood lymphocytes found that prominent changes occurred in γδ T cells and B cell subsets upon IFN-β treatment. This is the first report of a marked increase in Vδ1^−^Vδ2^−^Vγ9^−^ γδ T cells and decrease in Vδ2^+^ γδ T cells. A previous study reported a decrease in class-switched memory B cells by IFN-β [13, 14], which is confirmed in the present study. The increase of transitional B cells by IFN-β in RRMS [12] was also confirmed when analyzing RRMS patients separately. In IFN-β-treated MS patients, the percentages of naïve and transitional B cells were significantly higher in RRMS than in PMS. These findings are also comparable with previous reports describing an increase in naïve and transitional B cells and a decrease in memory B cells by IFN-β treatment in MS consisting of exclusively or mostly RRMS patients [12–14, 22]. It was also reported that the percentage of naïve T cells tended to decrease and that of memory T cells tended to increase in SPMS compared with RRMS [23]. Such differences by MS subtype are partly explained by the higher thymic export of naïve CD4^+^ T cells in RRMS than in PPMS [24]. In addition, naïve T cells were negatively associated and memory T cells were positively associated with age [23, 25]. The higher age at examination in PMS than RRMS in our cohort may also be partly responsible for the increase of effector memory T cells and decrease of naïve T cells in PMS. Importantly, effects of IFN-β on Vδ1^−^Vδ2^−^Vγ9^−^ γδ T cells were observed, regardless of the clinical subtype including RRMS and PMS. In addition, these changes in Vδ1^−^Vδ2^−^Vγ9^−^ γδ T cells were not observed in untreated MS patients even after stratification to NEDA and EDA groups, further supporting the relationship of changes in Vδ1^−^Vδ2^−^Vγ9^−^ γδ T cell percentages by IFN-β treatment.

When we designed the present study, we anticipated an improvement in the low percentages of Vδ2^+^Vγ9^+^ γδ T cells observed in untreated MS [10] by IFN-β treatment; however, we unexpectedly found that IFN-β increased Vδ1^−^Vδ2^−^Vγ9^−^ cell percentages in γδ T cells but did not reverse the decrease of Vδ2^+^Vγ9^+^ cells nor the loss of a positive correlation between Vδ2^+^Vγ9^+^ γδ T cells and Treg cells seen in HCs. Vδ1^−^Vδ2^−^Vγ9^−^ γδ T cells in MS had a negative correlation with class-switched memory B cells, which was specifically reduced by IFN-β treatment. In the super-responder (NEDA) group of IFN-β-treated MS, Vδ1^−^Vδ2^−^Vγ9^−^ γδ T cells had a negative correlation with EDSS scores. These findings suggest that Vδ1^−^Vδ2^−^Vγ9^−^ γδ T cells upregulated by IFN-β play a protective role against MS and that Vδ2^+^Vγ9^+^ γδ T cells may not play a major role in the treatment effects of IFN-β.

The majority of Vδ1^−^Vδ2^−^ γδ T cells express the Vδ3 TCR chain [26, 27]. Vδ3 γδ T cells are normally the third most common γδ T cell subset in peripheral blood lymphocytes and account for only ~ 0.5% of circulating T cells [26, 28], although they are enriched in the gut [29] and liver [28]. Vδ3 γδ T cells recognize human leukocyte antigen (HLA)-A2 [30] and CD1d [26], although the specific antigen molecules involved remain unknown. Upon activation, Vδ3 γδ T cells produce IFN-γ, IL-4, and IL-17, thereby promoting Th1, Th2, and Th17 cell differentiation, respectively [31]. Expansion of Vδ1^−^Vδ2^−^ or Vδ3 γδ T cells in the peripheral blood has seldom been reported; indeed, it was only observed upon cytomegalovirus reactivation after renal and stem cell transplantation [32, 33]. An alteration of Vδ1^−^Vδ2^−^ or Vδ3 γδ T cells has not been described in MS. Among the Vδ1^−^Vδ2^−^ cell subset increased by IFN-β treatment, Vδ1^−^Vδ2^−^Vγ9^−^ cells were increased but Vδ1^−^Vδ2^−^Vγ9^+^ cells were decreased. Vδ1^−^Vδ2^−^Vγ9^−^ cells had a negative correlation with EDSS scores, whereas Vδ1^−^Vδ2^−^Vγ9^+^ cells had a positive correlation with them. Because Vγ9^+^ subsets are functionally distinct from their Vγ9^−^ counterparts [34], these findings suggest that Vδ1^−^Vδ2^−^Vγ9^−^ cells but not Vδ1^−^Vδ2^−^Vγ9^+^ cells are related to the beneficial effects of IFN-β on MS.

Although the exact roles of Vδ3 γδ T cells under normal physiological conditions remain to be elucidated, they are assumed to have similar immunomodulatory functions to invariant natural killer T (iNKT) cells that recognize glycolipid antigens presented by CD1d molecules [35, 36]. In various animal models of autoimmune/inflammatory diseases, including experimental autoimmune encephalomyelitis, an animal model of MS, iNKT cells prevent inflammation [35–38]. Likewise, Vδ3 γδ T cells regulated adaptive immunity via the production of multiple cytokines [26]. In our study, in Vδ1^−^Vδ2^−^ cells, IFN-γ-producing cells were significantly decreased while IFN-γ^−^IL-17^−^ cells were increased in the IFN-β-treated MS patients. Thus, it is possible that Vδ1^−^Vδ2^−^Vγ9^−^ cells producing cytokines other than IFN-γ and IL-17 are associated with protection against MS by immunomodulatory effects in IFN-β-treated patients. The specific cytokines and functions related to these cells should be investigated in future studies.

Interestingly, we found a negative correlation of Vδ1^−^Vδ2^−^Vγ9^−^ cells with class-switched memory B cells in MS patients. In MS, the importance of B cells has become increasingly evident by the recent clinical trial results showing anti-CD20 monoclonal antibodies (rituximab, ocrelizumab, and ofatumumab) targeting B cells, but not plasma cells, are highly effective at reducing relapse and disability progression in MS [39–41]. Because the number of B cells but not the total antibody level decreased in parallel with the decrease in relapse rate [39–41], it is considered that B cell-T cell interactions, such as antigen presentation and proinflammatory cytokine secretion by B cells, are crucial in MS pathogenesis. In the present study, we found that IFN-β treatment markedly decreased class-switched memory B cells, which is consistent with previous reports demonstrating that IFN-β specifically reduced pathogenic memory B cells in MS [13, 14].

In our study, among IFN-β users, a positive correlation of class-switched memory B cells with EDSS scores was evident in the EDA group showing a wide range of responses. Presumably, the reduction of class-switched memory B cells by IFN-β treatment is partly responsible for the beneficial effects of IFN-β. The positive correlation between class-switched memory B cells and EDSS scores was not observed in super-responders to IFN-β (NEDA group), possibly because of plateau effects. Vδ3 γδ T cells induce the maturation and IgM secretion of B cells but do not induce class switching to IgG, IgA, or IgE [31]. Rather, Vδ3 γδ T cells exerted cytotoxic functions against CD1d-bearing target cells upon the recognition of CD1d molecules [26, 33]. Because B cells express high surface levels of CD1d [42, 43], they might be a target of Vδ3 γδ T cells. In addition, we found a negative correlation between Vδ1^−^Vδ2^−^Vγ9^−^ cells and class-switched memory B cells in MS patients, indicating that Vδ1^−^Vδ2^−^Vγ9^−^ cells might contribute to the attenuation of disease activity by killing class-switched memory B cells that are pathogenic in MS.

The present study had several limitations. First, we did not use an anti-Vδ3 TCR antibody for immunophenotyping because of the unavailability of a specific antibody. Although the majority of Vδ1^−^Vδ2^−^Vγ9^−^ cells harbor Vδ3 TCR [26, 27], future studies using anti-Vδ3 TCR antibody would be desirable to confirm our results. Second, the characterization of Vδ1^−^Vδ2^−^Vγ9^−^ cells was insufficient. More extensive assays on cytokine production and functional assays for the cytotoxicity of Vδ1^−^Vδ2^−^Vγ9^−^ cells are necessary to elucidate the roles of these cells. Third, the mechanism of how IFN-β increases the number of Vδ1^−^Vδ2^−^Vγ9^−^ cells remains unclear. However, Vδ3 γδ T cells have been shown to expand upon reactivation of chronic viral infections, such as cytomegalovirus [32, 33], which involve innate immune responses including type I IFN [44–46]. Thus, IFN-β may facilitate the expansion of Vδ1^−^Vδ2^−^Vγ9^−^ cells by a mechanism similar to that during persistent viral infection. Fourth, we did not examine anti-IFN-β neutralizing antibody status in the present study. In our cohort, IFN-β-1a was administered to 11 patients and IFN-β-1b to ten patients. Anti-IFN-β neutralizing antibodies are reported to develop for IFN-β-1a in approximately 5% and for IFN-β-1b in approximately 25% of MS patients [47]; therefore, two to three patients on IFN-β in our cohort may have had anti-IFN-β neutralizing antibodies. Thus, 15–23% of the 13 EDA patients may have possessed them at the time of the flow cytometry, which may have influenced the comparison between NEDA and EDA groups. However, the percentages of Vδ1^−^Vδ2^−^Vγ9^−^ γδ T cells were not significantly different between the EDA and NEDA groups while the EDA group still demonstrated a significantly higher percentage of Vδ1^−^Vδ2^−^Vγ9^−^ γδ T cells than the HC group. Therefore, we believe that the potential effects of anti-IFN-β neutralizing antibodies on γδ T cell subsets would not severely distort the present results, although this point should be investigated in future studies. Another limitation of this study is the absence of serum cytokine concentrations. We focused on specific immune cells, especially γδ T cells, and their cytokine production. γδ T cells comprise < 5% of lymphocytes in the peripheral blood [3], and the number of γδ T cells producing each cytokine is inconsistent with serum cytokine levels [48]. Given that serum cytokine concentration does not provide information about the source cell types, we did not measure serum cytokine levels in each patient. The correlation between serum cytokine concentrations and γδ T subsets should be studied in the future. Finally, because the numbers of enrolled patients in the NEDA and EDA groups consisting of IFN-β-treated MS patients were relatively small in this study, our findings should be confirmed in a future study using a larger sample size.

## Conclusions

Extensive flow cytometric immunophenotyping of peripheral blood lymphocytes from untreated and persistently IFN-β-treated MS patients revealed that the long-term usage of IFN-β increased the percentages of Vδ1^−^Vδ2^−^Vγ9^−^ γδ T cells and decreased the percentages of class-switched memory B cells without a major influence on other T and B lymphocyte subsets, which may contribute to the attenuation of disease activity. Because increased Vδ1^−^Vδ2^−^Vγ9^−^ cells were associated with a better outcome, especially in MS patients fulfilling NEDA under IFN-β treatment, Vδ1^−^Vδ2^−^Vγ9^−^ cells might be a target for the therapeutic immunomodulation of MS.

## Additional files

Additional file 1: **Table S1.** Clinical demographics of IFN-β-treated MS patients in the NEDA and EDA groups. **Table S2.** Clinical demographics of untreated MS patients in the NEDA and EDA groups. **Table S3.** Comparison of clinical demographics between RRMS and PMS patients. **Table S4.** Comparison of the percentages of CD4^+^ T cell subsets between the untreated MS group, IFN-β-treated MS group and healthy controls. **Table S5.** Comparison of the percentages of CD8^+^ T cell subsets between the untreated MS group, IFN-β-treated MS group and healthy controls. **Table S6.** Comparison of the percentages of cytokine-producing αβ T cell subsets between the untreated MS group, IFN-β-treated MS group and healthy controls. **Table S7.** Comparison of the percentages of cytokine-producing γδ T cell subsets in IFN-β-treated MS patients stratified to the NEDA or EDA groups. **Table S8.** Comparison of the percentages of γδ T cell subsets in untreated MS patients stratified to the NEDA or EDA groups. **Table S9.** Comparison of the percentages of B cell subsets in untreated MS patients stratified to the NEDA or EDA groups. **Table S10.** Comparison of the percentages of γδ T cell subsets between RRMS and PMS patients. **Table S11.** Comparison of the percentages of CD4^+^ T cell subsets between RRMS and PMS patients. **Table S12.** Comparison of the percentages of CD8^+^ T cell subsets between RRMS and PMS patients. **Table S13.** Comparison of the percentages of B cell subsets between RRMS and PMS patients **Table S14.** Comparison of the percentages of cytokine-producing γδ T cell subsets between RRMS and PMS patients. **Table S15.** Comparison of the percentages of cytokine-producing αβ T cell subsets between RRMS and PMS patients. (PDF 568 kb)

Additional file 2: **Figure S1.** Immunophenotyping gating strategy for γδ T cells and their comparison between HCs and patients with MS. **Figure S2.** Correlations between the percentages of γδ T cell subsets and disability in untreated MS patients. **Figure S3.** Correlations of memory B cell proportions with disability in untreated MS patients. (PDF 814 kb)

## Data Availability

The datasets generated and/or analyzed during the present study will be available from the corresponding author upon reasonable request based on the guidelines of the Ethics Committee of Kyushu University.

## Abbreviations

CNS: Central nervous system;
CNV: Copy number variation;
DMT: Disease-modifying therapy;
EDA: Evidence of disease activity;
EDSS: Expanded Disability Status Scale;
GM-CSF: Granulocyte macrophage colony-stimulating factor;
HCs: Healthy controls;
HLA: Human leukocyte antigen;
IFN: Interferon;
IL: Interleukin;
iNKT: Invariant natural killer T;
IQR: Interquartile range;
MS: Multiple sclerosis;
MSSS: Multiple Sclerosis Severity Score;
NEDA: No-evidence of disease activity;
PMS: Progressive multiple sclerosis;
RRMS: Relapsing-remitting multiple sclerosis;
TCR: T cell receptor;
Th: Helper T;
Treg: Regulatory T
