# Supplementary material for: Long-term use of interferon-β in multiple sclerosis increases Vδ1−Vδ2−Vγ9− γδ T cells that are associated with a better outcome
Source: J Neuroinflammation. 2019 Sep 13;16:179. doi: 10.1186/s12974-019-1574-5 (PMC6743159; doi:10.1186/s12974-019-1574-5)
Supplement: Supplementary file 1 — Table S1. Clinical demographics of IFN-β-treated MS patients in the NEDA and EDA groups. Table S2. Clinical demographics of untreated MS patients in the NEDA and EDA groups. Table S3. Comparison of clinical demographics between RRMS and PMS patients. Table S4. Comparison of the percentages of CD4+ T cell subsets between the untreated MS group, IFN-β-treated MS group and healthy controls. Table S5. Comparison of the percentages of CD8+ T cell subsets between the untreated MS group, IFN-β-treated MS group and healthy controls. Table S6. Comparison of the percentages of cytokine-producing αβ T cell subsets between the untreated MS group, IFN-β-treated MS group and healthy controls. Table S7. Comparison of the percentages of cytokine-producing γδ T cell subsets in IFN-β-treated MS patients stratified to the NEDA or EDA groups. Table S8. Comparison of the percentages of γδ T cell subsets in untreated MS patients stratified to the NEDA or EDA groups. Table S9. Comparison of the percentages of B cell subsets in untreated MS patients stratified to the NEDA or EDA groups. Table S10. Comparison of the percentages of γδ T cell subsets between RRMS and PMS patients. Table S11. Comparison of the percentages of CD4+ T cell subsets between RRMS and PMS patients. Table S12. Comparison of the percentages of CD8+ T cell subsets between RRMS and PMS patients. Table S13. Comparison of the percentages of B cell subsets between RRMS and PMS patients Table S14. Comparison of the percentages of cytokine-producing γδ T cell subsets between RRMS and PMS patients. Table S15. Comparison of the percentages of cytokine-producing αβ T cell subsets between RRMS and PMS patients. (PDF 568 kb) [file 12974_2019_1574_MOESM1_ESM.pdf]

Table S1 Clinical demographics of IFN-β-treated MS patients in the NEDA and EDA groups

	MS w/IFN-β		HCs (n = 44)	*p* value		
	**NEDA (n = 8)**	**EDA (n = 13)**		**NEDA vs. HCs**	**EDA vs. HCs**	**NEDA vs. EDA**
Female, *n* (%)	6 (75.0)	9 (69.2)	27 (61.4)	NS	NS	NS
Age at examination, years	47.0 [44.5-53.8]	44.0 [41.0-46.5]	35.0 [32.3-43.8]	0.004	0.010	NS
Age at disease onset, years	30.5 [25.5-34.8]	27.0 [23.5-34.0]	–	–	–	NS
Disease duration at INF-β initiation, years	6.83 [1.38-10.3]	6.50 [2.29-12.9]	–	–	–	NS
Disease duration at examination, years	15.8 [9.00-23.9]	15.2 [10.7-20.6]	–	–	–	NS
Subtype (RRMS / SPMS / PPMS), *n* (%)	7/1/0 (87.5/12.5/0.0)	6/6/1 (46.2/46.2/7.7)	–	–	–	NS
EDSS score at IFN-β initiation	3.0 [1.25-6.13]	2.5 [1.75-5.25]	–	–	–	NS
EDSS score at examination	2.0 [1.13-2.75]	4.5 [2.0-6.25]	–	–	–	NS (0.062)
ΔEDSS	−0.5 [−1.5-0.0]	1.0 [0.25-2.5]	–	–	–	0.003
MSSS at IFN-β initiation	4.89 [2.86-8.38]	6.00 [2.15-7.19]	–	–	–	NS
MSSS at examination	1.84 [0.57-3.65]	3.52 [1.99-7.39]	–	–	–	NS (0.060)
ΔMSSS	−2.69 [−5.16-−1.16]	−0.20 [−1.69-0.96]				0.013
Years of IFN-β treatment	9.50 [5.50-14.5]	7.00 [4.50-8.50]	–	–	–	NS

Values are the median [IQR] or count (%). ΔEDSS was calculated as the difference between EDSS scores at IFN-β initiation and at examination. ΔMSSS was the difference between MSSS at IFN-β initiation and at examination.

EDA = evidence of disease activity; EDSS = Expanded Disability Status Scale; HCs = healthy controls; IFN-β = interferon-β; IQR = interquartile ranges; MS = multiple sclerosis; MSSS = multiple sclerosis severity score; NEDA = no-evidence of disease activity; NS = not significant; PPMS = primary progressive MS; RRMS = relapsing-remitting MS; SPMS = secondary progressive MS; w/ = with.

Table S2 Clinical demographics of untreated MS patients in the NEDA and EDA groups

	**Untreated MS**			***p* value**		
	**NEDA (*n* = 19)**	**EDA (*n* = 13)**	**HCs (*n* = 44)**	**NEDA vs. HCs**	**EDA vs. HCs**	**NEDA vs. EDA**
Female, *n* (%)	16 (84.2)	12 (92.3)	27 (61.4)	NS (0.086)	0.044	NS
Age at examination, years	52.0 [38.0-62.0]	49.0 [35.5-55.5]	35.0 [32.3-43.8]	<0.001	NS (0.054)	NS
Age at disease onset, years	31.0 [24.0-44.0]	31.0 [24.5-36.5]	–	–	–	NS
Disease duration at examination, years	13.9 [8.2-26.9]	13.0 [4.6-21.0]	–	–	–	NS
Subtype (RRMS/SPMS/PPMS), *n* (%)	14/3/2 (73.7/15.8/10.5)	10/2/1 (76.9/15.4/7.7)	–	–	–	NS
EDSS score at 2 years before examination*	2.0 [0.0-4.0]	1.0 [0.0-5.4]	–	–	–	NS
EDSS score at examination	2.0 [0.0-4.0]	2.0 [1.0-5.5]	–	–	–	NS
ΔEDSS*	0.0 [0.0-0.0]	1.00 [1.0-2.5]	–	–	–	<0.001
MSSS at 2 years before examination*	2.13 [0.25-5.44]	1.27 [0.06-6.16]	–	–	–	NS
MSSS at examination	1.80 [0.21-5.16]	2.85 [0.88-8.89]	–	–	–	NS
ΔMSSS*	−0.09 [−0.62-−0.03]	1.18 [0.33-1.44]	–	–	–	<0.001

Values are the median [IQR] or count (%). ΔEDSS was calculated as the difference between EDSS scores at 2 years before examination and at examination. ΔMSSS was the difference between MSSS at 2 years before examination and at examination.

*The data of 5 patients are missing because of a lack of data before the onset.

EDA = evidence of disease activity; EDSS = Expanded Disability Status Scale; HCs = healthy controls; IFN-β = interferon-β; IQR = interquartile ranges; MS = multiple sclerosis; MSSS = multiple sclerosis severity score; NEDA = no-evidence of disease activity; NS = not significant; PPMS = primary progressive MS; RRMS = relapsing-remitting MS; SPMS = secondary progressive MS.

Table S3 Comparison of clinical demographics between RRMS and PMS patients

	**Untreated MS**			**MS w/IFN-β**		
	**RRMS (*n* = 26)**	**PMS (*n* = 9)**	***p* value**	**RRMS (*n* = 13)**	**PMS (*n* = 8)**	***p* value**
Female, *n* (%)	23 (88.5)	7 (77.8)	NS	9 (69.2)	6 (75.0)	NS
Age at examination, years	45.0 [36.5-54.0]	56.0 [46.5-67.5]	0.043	46.0 [43.0-47.0]	44.0 [40.8-51.5]	NS
Age at disease onset, years	31.0 [27.8-36.0]	29.0 [19.0-55.0]	NS	30.0 [25.0-34.0]	26.0 [21.0-36.8]	NS
Disease duration at examination, years	13.0 [7.6-18.3]	20.9 [10.7-28.1]	NS (0.086)	15.4 [11.1-21.7]	15.2 [10.2-20.8]	NS
EDSS score at examination	1.5 [0.0-3.0]	7.0 [6.25-7.25]	<0.001	2.0 [1.5-2.5]	6.25 [5.63-6.88]	<0.001
MSSS at examination	1.57 [0.24-2.99]	7.97 [6.56-8.60]	<0.001	1.64 [0.61-2.88]	7.24 [6.78-7.60]	<0.001
Disease duration at IFN-β initiation, years	–	–	–	7.1 [1.7-12.9]	5.8 [1.9-11.5]	NS
EDSS score at IFN-β initiation	–	–	–	2.0 [1.0-3.5]	4.0 [2.5-6.0]	NS (0.074)
MSSS at IFN-β initiation	–	–	–	3.69 [1.32-5.62]	7.19 [6.04-7.74]	0.015
Years of IFN-β treatment	–	–	–	7.0 [4.0-9.5]	8.5 [6.3-13.8]	NS

Values are the median [IQR] or count (%).

EDSS = Expanded Disability Status Scale; IFN-β = interferon-β; IQR = interquartile ranges; MS = multiple sclerosis; MSSS = MS severity score; NS = not significant; PMS = progressive MS; RRMS = relapsing-remitting MS; w/ = with.

Table S4 Comparison of the percentages of $CD4^+$ T cell subsets between the untreated MS group, IFN-β-treated MS group and healthy controls

In total $CD4^+$ T cells	Untreated MS (*n* = 35)	MS w/ IFN-β (*n* = 21)	HCs (*n* = 44)	*p* value (K-W test)	p^{adj} value		
					Untreated MS vs. HCs	MS w/ IFN-β vs. HCs	Untreated MS vs. MS w/ IFN-β
Tnaive ($CCR7^+CD45RA^+$)	45.3 [38.8-59.4]	51.2 [41.2-58.8]	49.4 [36.1-56.9]	NS	–	–	–
Tcm ($CCR7^+CD45RA^–$)	27.9 [21.7-33.7]	26.7 [21.0-32.0]	25.7 [23.2-33.6]	NS	–	–	–
Tem ($CCR7^–CD45RA^–$)	19.2 [12.5-24.7]	16.4 [13.9-26.8]	18.8 [15.3-28.8]	NS	–	–	–
Teff ($CCR7^–CD45RA^+$)	2.82 [1.74-3.87]	2.87 [2.20-4.58]	2.51 [1.99-3.67]	NS	–	–	–
Activated T ($HLA\text{-}DR^+$)	1.96 [1.50-3.07]	2.36 [1.72-3.02]	2.81 [1.59-3.57]	NS	–	–	–
Treg ($CD25^+CD127^{low/–}$)	4.41 [2.83-5.95]	3.80 [3.50-5.82]	5.97 [4.34-6.87]	0.012	NS	NS (0.077)	NS

Values are the median [IQR]. Percentages of each population in total $CD4^+$ T cells are shown.

p values (K-W test) were obtained by Kruskal-Wallis analyses, and if they were statistically significant, then p^{adj} values were calculated using multivariate linear regression analyses adjusted for age and sex.

HCs = healthy controls; IFN-β = interferon-β; IQR = interquartile ranges; K-W = Kruskal-Wallis; MS = multiple sclerosis; NS = not significant; Tcm = central memory T cells; Teff = effector T cells; Tem = effector memory T cells; Tnaive = naïve T cells; Treg = regulatory T cells; w/ = with.

Table S5 Comparison of the percentages of CD8$^+$ T cell subsets between the untreated MS group, IFN-β-treated MS group and healthy controls

In total CD8$^+$ T cells	Untreated MS (*n* = 35)	MS w/ IFN-β (*n* = 21)	HCs (*n* = 44)	*p* value (K-W test)	p^{adj} value		
					Untreated MS vs. HCs	MS w/ IFN-β vs. HCs	Untreated MS vs. MS w/ IFN-β
Tnaive (CCR7$^+$CD45RA$^+$)	28.1 [17.7-53.8]	46.0 [28.6-64.3]	44.4 [33.6-54.2]	0.035	NS	NS	0.031
Tcm (CCR7$^+$CD45RA$^-$)	5.14 [3.04-9.04]	8.68 [5.51-12.0]	5.55 [3.72-7.58]	0.017	NS	NS	0.013
Tem (CCR7$^-$CD45RA$^-$)	37.6 [26.7-46.6]	26.7 [16.9-45.3]	29.2 [23.9-44.0]	NS	–	–	–
Teff (CCR7$^-$CD45RA$^+$)	15.9 [8.70-25.2]	11.6 [6.68-17.4]	12.8 [8.16-22.8]	NS	–	–	–
Activated T (HLA-DR$^+$)	4.27 [2.72-6.83]	4.55 [2.79-6.58]	3.63 [2.07-6.17]	NS	–	–	–

Values are the median [IQR]. Percentages of each population in total CD8$^+$ T cells are shown.

p values (K-W test) were obtained by Kruskal-Wallis analyses, and if they were statistically significant, then p^{adj} values were calculated using multivariate linear regression analyses adjusted for age and sex.

HCs = healthy controls; IFN-β = interferon-β; IQR = interquartile ranges; K-W = Kruskal-Wallis; MS = multiple sclerosis; NS = not significant; Tcm = central memory T cells; Teff = effector T cells; Tem = effector memory T cells; Tnaive = naïve T cells; w/ = with.

Table S6 Comparison of the percentages of cytokine-producing αβ T cell subsets between the untreated MS group, IFN-β-treated MS group and healthy controls

	**Untreated MS (*n* = 35)**	**MS w/ IFN-β (*n* = 21)**	**HCs (*n* = 44)**	***p* value (K-W test)**	**p^{adj} value**		
					**Untreated MS vs. HCs**	**MS w/ IFN-β vs. HCs**	**Untreated MS vs. MS w/ IFN-β**
**In CD4$^+$ T cells**							
IL-17A$^+$	0.24 [0.16-0.58]	0.37 [0.27-0.61]	0.55 [0.25-1.11]	0.004	0.017	NS	NS
IFN-γ$^+$	5.77 [2.04-8.24]	6.70 [2.38-10.3]	9.42 [4.59-14.6]	NS (0.052)	–	–	–
IL-4$^+$	1.70 [0.83-2.91]	1.64 [1.04-2.69]	2.76 [1.67-4.31]	0.018	0.011	NS	NS
GM-CSF$^+$	1.36 [0.76-3.19]	1.90 [1.56-3.71]	5.40 [1.19-7.91]	0.002	0.002	0.034	NS
IL-17A$^+$IFN-γ$^+$	0.03 [0.01-0.08]	0.04 [0.02-0.08]	0.06 [0.02-0.14]	0.039	NS (0.093)	NS	NS
IL-17A$^+$GM-CSF$^+$	0.04 [0.01-0.08]	0.05 [0.03-0.11]	0.15 [0.07-0.32]	<0.001	0.010	NS (0.081)	NS
**In CD8$^+$ T cells**							
IL-17A$^+$	0.17 [0.08-0.28]	0.24 [0.14-0.39]	0.16 [0.10-0.31]	NS	–	–	–
IFN-γ$^+$	18.7 [10.1-37.6]	15.6 [4.97-33.0]	27.2 [16.1-38.3]	NS	–	–	–
IL-17A$^+$IFN-γ$^+$	0.05 [0.02-0.10]	0.06 [0.02-0.07]	0.07 [0.02-0.14]	NS	–	–	–

Values are the median [IQR]. Percentages of each population are shown.

p values (K-W test) were obtained by Kruskal-Wallis analyses, and if they were statistically significant, then p^{adj} values were calculated using multivariate linear regression analyses adjusted for age and sex.

GM-CSF = granulocyte macrophage colony-stimulating factor; HCs = healthy controls; IFN = interferon; IL = interleukin; IQR = interquartile ranges; K-W = Kruskal-Wallis; MS = multiple sclerosis; NS = not significant; w/ = with.

Table S7 Comparison of the percentages of cytokine-producing γδ T cell subsets in IFN-β-treated MS patients stratified to the NEDA or EDA groups

	MS w/IFN-β				p^{adj} value		
	**NEDA (n = 8)**	**EDA (n = 13)**	**HCs (n = 44)**	**p value (K-W test)**	**NEDA vs. HCs**	**EDA vs. HCs**	**NEDA vs. EDA**
**In Vδ1$^+$ γδT cells**							
IL-17A$^+$	0.58 [0.10-1.04]	0.27 [0.00-1.20]	0.18 [0.01-0.72]	NS	–	–	–
IFN-γ$^+$	35.2 [17.7-56.9]	25.0 [5.75-34.3]	45.8 [24.4-60.8]	0.015	NS	0.001	NS (0.070)
IL-17A$^+$IFN-γ$^+$	0.21 [0.04-0.75]	0.11 [0.00-0.55]	0.08 [0.00-0.31]	NS	–	–	–
IL-17A$^-$IFN-γ$^-$	64.8 [42.9-82.0]	75.0 [65.3-93.2]	54.1 [39.1-75.5]	0.014	NS	0.001	NS (0.074)
**In Vδ2$^+$ γδT cells**							
IL-17A$^+$	0.00 [0.00-2.10]	0.00 [0.00-0.82]	0.13 [0.01-0.52]	NS	–	–	–
IFN-γ$^+$	39.9 [23.1-74.7]	32.4 [18.8-58.7]	83.6 [69.1-92.7]	<0.001	0.003	<0.001	NS
IL-17A$^+$IFN-γ$^+$	0.00 [0.00-1.56]	0.00 [0.00-0.18]	0.11 [0.01-0.42]	NS	–	–	–
IL-17A$^-$IFN-γ$^-$	60.1 [25.0-76.9]	67.6 [40.8-78.9]	16.0 [7.30-30.9]	<0.001	0.003	<0.001	NS
**In Vδ1$^-$Vδ2$^-$ γδT cells**							
IL-17A$^+$	1.03 [0.39-2.13]	0.79 [0.31-1.87]	1.24 [0.66-2.81]	NS	–	–	–
IFN-γ$^+$	31.1 [14.6-52.8]	14.2 [8.88-27.3]	47.1 [32.5-58.2]	<0.001	NS	<0.001	0.033
IL-17A$^+$IFN-γ$^+$	0.44 [0.19-0.80]	0.34 [0.00-0.65]	0.35 [0.19-1.22]	NS	–	–	–
IL-17A$^-$IFN-γ$^-$	68.6 [46.4-84.7]	84.0 [72.2-88.6]	51.7 [41.3-66.9]	<0.001	NS	<0.001	0.038

Values are the median [IQR]. Percentages of each population are shown.

p values (K-W test) were obtained by Kruskal-Wallis analyses, and if they were statistically significant, then p^{adj} values were calculated using multivariate linear regression analyses adjusted for age and sex.

EDA = evidence of disease activity; HCs = healthy controls; IFN = interferon; IL = interleukin; IQR = interquartile ranges; K-W = Kruskal-Wallis; MS = multiple sclerosis; NEDA = no-evidence of disease activity; NS = not significant; w/ = with.

Table S8 Comparison of the percentages of γδ T cell subsets in untreated MS patients stratified to the NEDA or EDA groups

	**Untreated MS**				p^{adj} **value**		
	**NEDA (*n* = 19)**	**EDA (*n* = 13)**	**HCs (*n* = 44)**	***p* value (K-W test)**	**NEDA vs. HCs**	**EDA vs. HCs**	**NEDA vs. EDA**
$V\delta1^+$	29.0 [14.0-71.5]	31.9 [17.5-51.8]	18.4 [11.6-34.2]	NS	–	–	–
$V\delta1^+V\gamma9^+$	4.00 [1.63-16.3]	3.91 [1.14-8.07]	2.15 [1.12-4.71]	NS	–	–	–
$V\delta1^+V\gamma9^-$	23.1 [10.2-42.3]	30.4 [15.0-41.4]	15.5 [8.47-31.5]	NS	–	–	–
$V\delta2^+$	38.5 [5.30-56.2]	29.9 [15.6-51.7]	54.9 [31.7-65.9]	0.024	NS	NS (0.076)	NS
$V\delta2^+V\gamma9^+$	37.9 [5.16-55.9]	29.8 [14.5-51.0]	54.3 [31.1-65.7]	0.021	NS	NS (0.073)	NS
$V\delta2^+V\gamma9^-$	0.19 [0.11-0.46]	0.14 [0.03-0.67]	0.08 [0.03-0.29]	NS	–	–	–
$V\delta1^-V\delta2^-$	21.1 [14.1-27.3]	28.2 [17.3-43.0]	22.8 [17.1-30.3]	NS	–	–	–
$V\delta1^-V\delta2^-V\gamma9^+$	1.61 [0.30-2.69]	1.10 [0.46-3.15]	2.42 [1.25-3.83]	NS (0.069)	–	–	–
$V\delta1^-V\delta2^-V\gamma9^-$	20.0 [11.0-25.7]	27.5 [17.2-36.9]	18.1 [10.7-26.4]	NS (0.065)	–	–	–

Values are the median [IQR]. Percentages of each population in total γδ T cells are shown.

p values (K-W test) were obtained by Kruskal-Wallis analyses, and if they were statistically significant, then p^{adj} values were calculated using multivariate linear regression analyses adjusted for age and sex.

EDA = evidence of disease activity; HCs = healthy controls; IFN-β = interferon-β; IQR = interquartile ranges; K-W = Kruskal-Wallis; MS = multiple sclerosis; NEDA = no-evidence of disease activity; NS = not significant.

Table S9 Comparison of the percentages of B cell subsets in untreated MS patients stratified to the NEDA or EDA groups

	**Untreated MS**				**p^{adj} value**		
	**NEDA (n = 19)**	**EDA (n = 13)**	**HCs (n = 44)**	**p value (K-W test)**	**NEDA vs. HCs**	**EDA vs. HCs**	**NEDA vs. EDA**
Naïve ($CD27^{-}IgD^{+}$)	46.3 [27.1-54.0]	53.8 [38.8-59.5]	51.8 [41.4-60.6]	NS	–	–	–
Memory ($CD27^{+}$)	13.4 [9.82-24.4]	19.9 [12.6-36.2]	22.7 [16.9-29.9]	0.024	NS	NS	NS
CS^{+} Memory ($CD27^{+}IgD^{-}$)	11.2 [8.84-22.0]	18.0 [11.1-32.7]	18.8 [14.4-25.2]	NS (0.060)	–	–	–
CS^{-} Memory ($CD27^{+}IgD^{+}$)	1.65 [0.72-2.68]	2.28 [1.86-3.80]	3.49 [2.49-4.67]	<0.001	0.043	NS	NS
Plasmablasts ($CD38^{high}CD20^{-}$)	0.25 [0.12-0.49]	0.61 [0.28-1.48]	0.37 [0.24-0.67]	0.030	NS	NS (0.064)	0.008
Transitional ($CD24^{high}CD38^{high}$)	3.16 [1.80-4.14]	2.50 [0.58-6.09]	3.14 [2.32-4.45]	NS	–	–	–

Values are the median [IQR]. Percentages of each population in total B cells are shown.

p values (K-W test) were obtained by Kruskal-Wallis analyses, and if they were statistically significant, then p^{adj} values were calculated using multivariate linear regression analyses adjusted for age and sex.

CS^{+} = class-switched; CS^{-} = non-class-switched; EDA = evidence of disease activity; HCs = healthy controls; IFN-β = interferon-β; IQR = interquartile ranges; K-W = Kruskal-Wallis; MS = multiple sclerosis; NEDA = no-evidence of disease activity; NS = not significant.

Table S10 Comparison of the percentages of γδ T cell subsets between RRMS and PMS patients

	**Untreated MS**			**MS w/IFN-β**		
	**RRMS (*n* = 26)**	**PMS (*n* = 9)**	***p* value**	**RRMS (*n* = 13)**	**PMS (*n* = 8)**	***p* value**
$V\delta1^+$	27.0 [13.3-47.4]	49.7 [16.8-77.2]	NS	29.6 [14.9-52.0]	24.5 [12.7-43.6]	NS
$V\delta1^+V\gamma9^+$	3.26 [0.93-5.31]	11.1 [2.64-20.9]	NS (0.062)	3.22 [1.88-6.70]	2.68 [1.52-3.90]	NS
$V\delta1^+V\gamma9^-$	22.5 [9.64-38.0]	36.7 [10.6-51.2]	NS	23.5 [9.18-42.7]	21.9 [11.4-35.3]	NS
$V\delta2^+$	37.0 [18.6-54.8]	7.34 [2.11-54.3]	NS	11.0 [3.75-24.2]	33.4 [11.2-44.5]	NS (0.076)
$V\delta2^+V\gamma9^+$	36.0 [18.0-54.6]	7.34 [1.87-54.0]	NS	10.6 [3.55-23.8]	32.2 [11.1-44.4]	NS (0.089)
$V\delta2^+V\gamma9^-$	0.20 [0.11-0.51]	0.06 [0.00-0.27]	NS	0.21 [0.11-0.72]	0.06 [0.00-0.20]	NS (0.099)
$V\delta1^-V\delta2^-$	26.2 [16.4-42.9]	22.7 [14.5-38.7]	NS	43.6 [22.8-63.4]	32.3 [27.7-43.2]	NS
$V\delta1^-V\delta2^-V\gamma9^+$	1.89 [0.38-3.57]	0.66 [0.10-1.31]	0.050	2.22 [0.33-2.82]	0.88 [0.25-1.48]	NS
$V\delta1^-V\delta2^-V\gamma9^-$	23.8 [15.4-34.8]	21.3 [13.8-37.0]	NS	41.4 [19.5-60.1]	31.7 [25.4-42.5]	NS

Values are the median [IQR]. Percentages of each population in total γδ T cells are shown.

IFN-β = interferon-β; IQR = interquartile ranges; MS = multiple sclerosis; NS = not significant; PMS = progressive MS; RRMS = relapsing-remitting MS; w/ = with.

Table S11 Comparison of the percentages of $CD4^+$ T cell subsets between RRMS and PMS patients

In total $CD4^+$ T cells	Untreated MS			MS w/IFN-β		
	**RRMS (*n* = 26)**	**PMS (*n* = 9)**	***p* value**	**RRMS (*n* = 13)**	**PMS (*n* = 8)**	***p* value**
Tnaive ($CCR7^+CD45RA^+$)	55.8 [43.4-66.2]	38.8 [32.9-41.8]	0.002	51.9 [44.4-57.1]	44.6 [33.2-60.6]	NS
Tcm ($CCR7^+CD45RA^–$)	25.3 [21.2-33.5]	30.4 [28.0-38.0]	NS	26.7 [19.4-34.1]	27.5 [21.7-31.6]	NS
Tem ($CCR7^–CD45RA^–$)	17.6 [12.1-22.1]	26.4 [20.8-35.0]	0.004	16.4 [14.7-19.0]	22.8 [10.7-34.7]	NS
Teff ($CCR7^–CD45RA^+$)	2.75 [1.73-3.52]	2.85 [1.77-7.60]	NS	2.87 [2.19-5.39]	2.76 [2.15-3.03]	NS
Activated T ($HLA\text{-}DR^+$)	1.86 [1.31-2.86]	2.55 [1.84-4.01]	NS (0.070)	2.36 [1.83-2.91]	2.05 [1.06-4.73]	NS
Treg ($CD25^+CD127^{low/–}$)	4.16 [2.39-5.85]	5.21 [4.05-6.12]	NS	3.83 [3.57-5.82]	3.66 [2.30-6.13]	NS

Values are the median [IQR]. Percentages of each population in total $CD4^+$ T cells are shown.

IFN-β = interferon-β; IQR = interquartile ranges; MS = multiple sclerosis; NS = not significant; PMS = progressive MS; RRMS = relapsing-remitting MS; Tcm = central memory T cells; Teff = effector T cells; Tem = effector memory T cells; Tnaive = naïve T cells; Treg = regulatory T cells; w/ = with.

Table S12 Comparison of the percentages of $CD8^+$ T cell subsets between RRMS and PMS patients

In total $CD8^+$ T cells	Untreated MS			MS w/IFN-β		
	**RRMS (*n* = 26)**	**PMS (*n* = 9)**	***p* value**	**RRMS (*n* = 13)**	**PMS (*n* = 8)**	***p* value**
Tnaive ($CCR7^+CD45RA^+$)	31.7 [19.6-58.2]	21.9 [6.77-28.7]	NS (0.052)	52.1 [43.0-64.3]	28.6 [16.4-70.9]	NS
Tcm ($CCR7^+CD45RA^–$)	5.22 [3.03-7.81]	3.45 [2.28-9.97]	NS	9.97 [7.87-13.1]	6.15 [3.41-11.0]	NS (0.076)
Tem ($CCR7^–CD45RA^–$)	36.4 [25.0-46.0]	43.5 [34.8-55.6]	NS	21.1 [16.9-30.6]	45.3 [14.5-64.8]	NS
Teff ($CCR7^–CD45RA^+$)	15.9 [7.62-26.5]	19.4 [12.0-41.1]	NS	11.6 [6.18-17.4]	12.7 [7.29-23.0]	NS
Activated T ($HLA\text{-}DR^+$)	4.11 [2.71-7.25]	4.27 [2.85-6.43]	NS	4.77 [4.12-6.58]	3.07 [1.67-6.43]	NS

Values are the median [IQR]. Percentages of each population in total $CD8^+$ T cells are shown.

IFN-β = interferon-β; IQR = interquartile ranges; MS = multiple sclerosis; NS = not significant; PMS = progressive MS; RRMS = relapsing-remitting MS; Tcm = central memory T cells; Teff = effector T cells; Tem = effector memory T cells; Tnaive = naïve T cells; Treg = regulatory T cells; w/ = with.

Table S13 Comparison of the percentages of B cell subsets between RRMS and PMS patients

	**Untreated MS**			**MS w/IFN-β**		
	**RRMS (*n* = 26)**	**PMS (*n* = 9)**	***p* value**	**RRMS (*n* = 13)**	**PMS (*n* = 8)**	***p* value**
Naïve ($CD27^{-}IgD^{+}$)	45.5 [27.7-56.3]	52.0 [29.6-60.7]	NS	55.6 [50.3-63.2]	35.1 [18.0-40.9]	<0.001
Memory ($CD27^{+}$)	17.3 [11.4-29.2]	11.3 [8.47-19.8]	NS	8.81 [6.12-12.5]	14.0 [8.42-23.8]	NS (0.065)
CS^{+} memory ($CD27^{+}IgD^{-}$)	15.3 [10.2-25.6]	10.7 [7.72-18.1]	NS	6.93 [5.31-11.3]	13.2 [7.48-20.9]	NS (0.060)
CS^{-} memory ($CD27^{+}IgD^{+}$)	2.27 [0.98-2.78]	1.65 [0.57-2.50]	NS	1.13 [0.89-1.67]	0.92 [0.64-2.86]	NS
Plasmablasts ($CD38^{high}CD20^{-}$)	0.28 [0.11-0.59]	0.49 [0.16-1.65]	NS	0.36 [0.21-0.47]	0.31 [0.28-1.14]	NS
Transitional ($CD24^{high}CD38^{high}$)	2.83 [1.88-6.00]	3.20 [0.48-7.00]	NS	6.36 [4.16-10.5]	1.52 [1.05-5.14]	0.015

Values are the median [IQR]. Percentages of each population in total B cells are shown.

CS^{+} = class-switched; CS^{-} = non-class-switched; IFN-β = interferon-β; IQR = interquartile ranges; MS = multiple sclerosis; NS = not significant; PMS = progressive MS; RRMS = relapsing-remitting MS; w/ = with.

Table S14 Comparison of the percentages of cytokine-producing γδ T cell subsets between RRMS and PMS patients

	Untreated MS			MS w/IFN-β		
	**RRMS (*n* = 26)**	**PMS (*n* = 9)**	***p* value**	**RRMS (*n* = 13)**	**PMS (*n* = 8)**	***p* value**
**In Vδ1^{+} γδ T cells**						
IL-17A^{+}	0.13 [0.00-0.41]	0.12 [0.00-0.50]	NS	0.54 [0.02-1.03]	0.33 [0.04-1.16]	NS
IFN-γ^{+}	35.9 [16.5-40.8]	36.6 [12.4-54.9]	NS	25.0 [7.14-45.2]	32.7 [11.9-37.9]	NS
IL-17A^{+}IFN-γ^{+}	0.03 [0.00-0.14]	0.00 [0.00-0.12]	NS	0.17 [0.00-0.66]	0.18 [0.00-0.58]	NS
IL-17A^{-}IFN-γ^{-}	64.1 [58.5-81.8]	63.4 [45.0-87.4]	NS	75.0 [54.8-91.9]	66.8 [62.1-87.7]	NS
**In Vδ2^{+} γδ T cells**						
IL-17A^{+}	0.07 [0.00-0.45]	0.00 [0.00-0.47]	NS	0.00 [0.00-2.63]	0.00 [0.00-1.15]	NS
IFN-γ^{+}	51.5 [12.7-85.1]	37.5 [6.33-62.4]	NS	38.1 [24.6-56.3]	49.2 [11.4-71.9]	NS
IL-17A^{+}IFN-γ^{+}	0.00 [0.00-0.29]	0.00 [0.00-0.06]	NS	0.00 [0.00-1.06]	0.00 [0.00-0.47]	NS
IL-17A^{-}IFN-γ^{-}	48.5 [14.9-87.3]	62.5 [36.8-93.2]	NS	61.9 [43.4-75.4]	50.4 [27.2-88.5]	NS
**In Vδ1^{-}Vδ2^{-} γδ T cells**						
IL-17A^{+}	0.63 [0.27-1.38]	0.53 [0.25-1.43]	NS	0.73 [0.40-1.72]	1.22 [0.20-1.93]	NS
IFN-γ^{+}	29.3 [14.0-40.7]	23.9 [6.52-41.7]	NS	15.8 [11.6-42.4]	24.8 [7.81-31.7]	NS
IL-17A^{+}IFN-γ^{+}	0.23 [0.03-0.53]	0.17 [0.00-0.51]	NS	0.34 [0.18-0.48]	0.65 [0.00-0.92]	NS
IL-17A^{-}IFN-γ^{-}	70.6 [58.9-85.6]	75.8 [57.9-93.5]	NS	83.9 [56.7-87.8]	75.1 [67.3-91.9]	NS

Values are the median [IQR]. Percentages of each population are shown.

IFN = interferon; IL = interleukin; IQR = interquartile ranges; MS = multiple sclerosis; NS = not significant; PMS = progressive MS; RRMS = relapsing-remitting MS; w/ = with.

Table S15 Comparison of the percentages of cytokine-producing αβ T cell subsets between RRMS and PMS patients

	Untreated MS			MS w/IFN-β		
	**RRMS (*n* = 26)**	**PMS (*n* = 9)**	***p* value**	**RRMS (*n* = 13)**	**PMS (*n* = 8)**	***p* value**
**In $CD4^+$ T cells**						
$IL\text{-}17A^+$	0.24 [0.15-0.61]	0.29 [0.11-0.47]	NS	0.39 [0.27-0.57]	0.33 [0.27-0.62]	NS
$IFN\text{-}\gamma^+$	4.42 [2.01-7.32]	8.20 [3.19-9.98]	NS	6.70 [2.38-8.74]	8.22 [2.57-16.0]	NS
$IL\text{-}4^+$	1.48 [0.80-2.81]	2.46 [1.06-3.85]	NS	1.64 [1.04-2.44]	2.12 [1.06-3.55]	NS
$GM\text{-}CSF^+$	1.47 [0.76-3.22]	1.36 [0.48-4.01]	NS	2.59 [1.78-4.12]	1.68 [1.28-2.13]	NS
$IL\text{-}17A^+IFN\text{-}\gamma^+$	0.03 [0.01-0.06]	0.03 [0.01-0.05]	NS	0.04 [0.02-0.09]	0.04 [0.02-0.08]	NS
$IL\text{-}17A^+GM\text{-}CSF^+$	0.04 [0.01-0.10]	0.04 [0.01-0.05]	NS	0.08 [0.03-0.15]	0.04 [0.02-0.05]	NS (0.089)
**In $CD8^+$ T cells**						
$IL\text{-}17A^+$	0.19 [0.10-0.37]	0.13 [0.06-0.17]	NS	0.25 [0.16-0.63]	0.18 [0.13-0.25]	NS
$IFN\text{-}\gamma^+$	16.8 [9.97-29.1]	37.6 [8.73-50.7]	NS	15.3 [4.97-20.9]	33.0 [7.12-41.5]	NS
$IL\text{-}17A^+IFN\text{-}\gamma^+$	0.05 [0.02-0.12]	0.04 [0.01-0.08]	NS	0.06 [0.02-0.13]	0.04 [0.03-0.06]	NS

Values are the median [IQR]. Percentages of each population are shown.

GM-CSF = granulocyte macrophage colony-stimulating factor; IFN = interferon; IL = interleukin; IQR = interquartile ranges; MS = multiple sclerosis; NS = not significant; PMS = progressive MS; RRMS = relapsing-remitting MS; w/ = with.
