# Supplementary material for: Long-term use of interferon-β in multiple sclerosis increases Vδ1−Vδ2−Vγ9− γδ T cells that are associated with a better outcome
Source: J Neuroinflammation. 2019 Sep 13;16:179. doi: 10.1186/s12974-019-1574-5 (PMC6743159; doi:10.1186/s12974-019-1574-5)
Supplement: Supplementary file 2 — Figure S1. Immunophenotyping gating strategy for γδ T cells and their comparison between HCs and patients with MS. Figure S2. Correlations between the percentages of γδ T cell subsets and disability in untreated MS patients. Figure S3. Correlations of memory B cell proportions with disability in untreated MS patients. (PDF 814 kb) [file 12974_2019_1574_MOESM2_ESM.pdf]

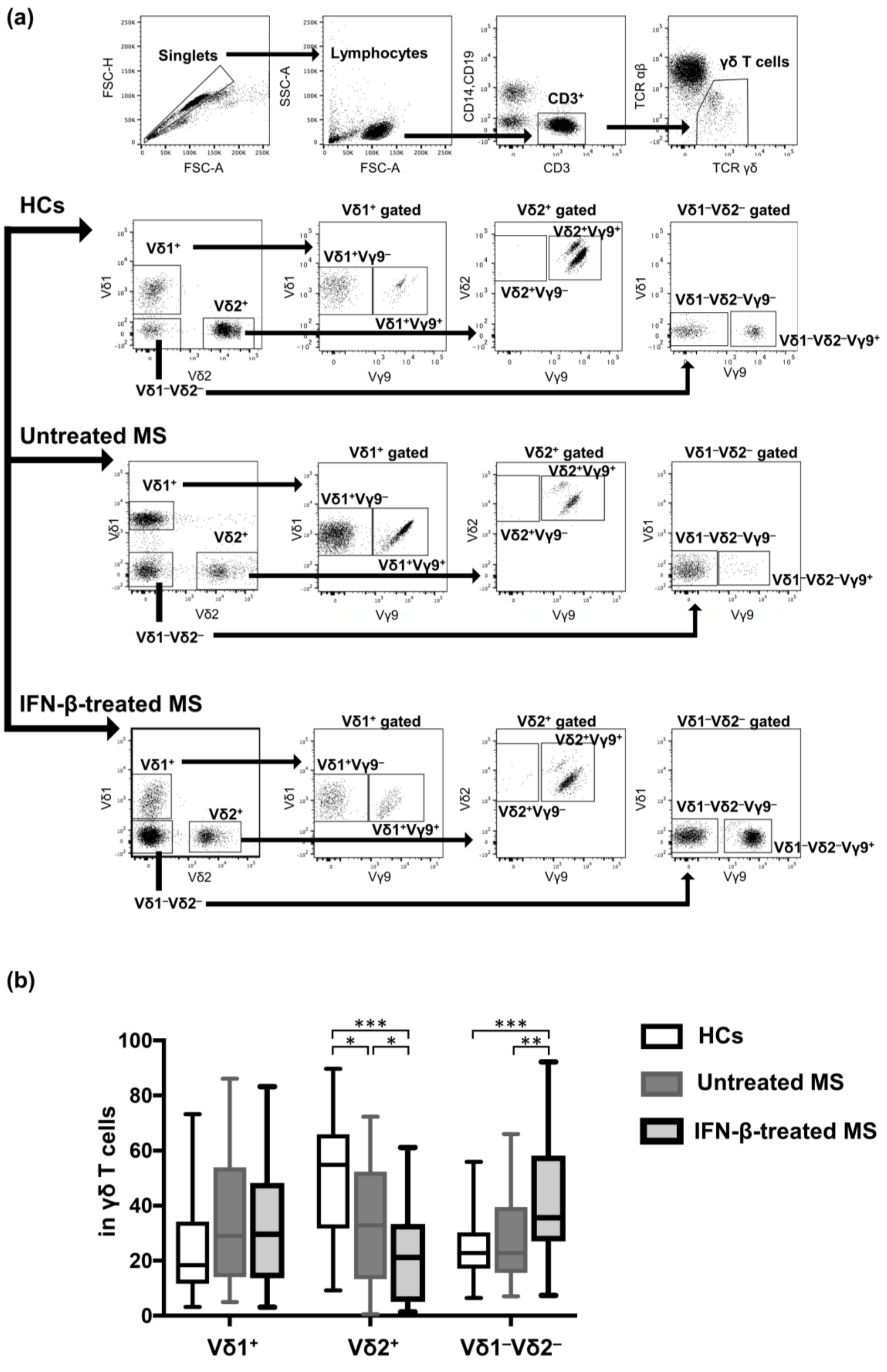

Figure S1 Immunophenotyping gating strategy for γδ T cells and their comparison between HCs and patients with MS.

(**a**) Gating strategies for the flow cytometric analysis of γδ T cells are shown. Peripheral blood mononuclear cells (PBMCs) were initially gated on singlet cells by forward-scatter (FSC)-area (FSC-A) and FSC-height (FSC-H), and then lymphocytes were gated by FSC-A and side-scatter (SSC)-area (SSC-A). γδ T cells were gated from $CD3^+$ T lymphocytes by TCRγδ and TCRαβ, and classified by the presence of Vδ1 and Vδ2 into $V\delta1^+V\delta2^-$, $V\delta1^-V\delta2^+$ and $V\delta1^-V\delta2^-$ γδ T cells, which were then examined for the expression of Vγ9. Representative data of HCs, untreated MS patients and IFN-β-treated MS patients are shown. (**b**) Comparison of the percentages of $V\delta1^+$, $V\delta2^+$ and $V\delta1^-V\delta2^-$ cells in γδ T cells between HCs, untreated MS patients and IFN-β-treated MS patients. Boxes depict the median and IQR, and upper/lower whiskers extend from the hinge toward the largest/smallest values. p^{adj} values were obtained by multivariate linear regression analyses adjusting for age and sex (* $p^{adj} < 0.05$, ** $p^{adj} < 0.01$ and *** $p^{adj} < 0.001$).
Abbreviations: IFN-β = interferon-β; MS = multiple sclerosis; TCR = T cell receptor.

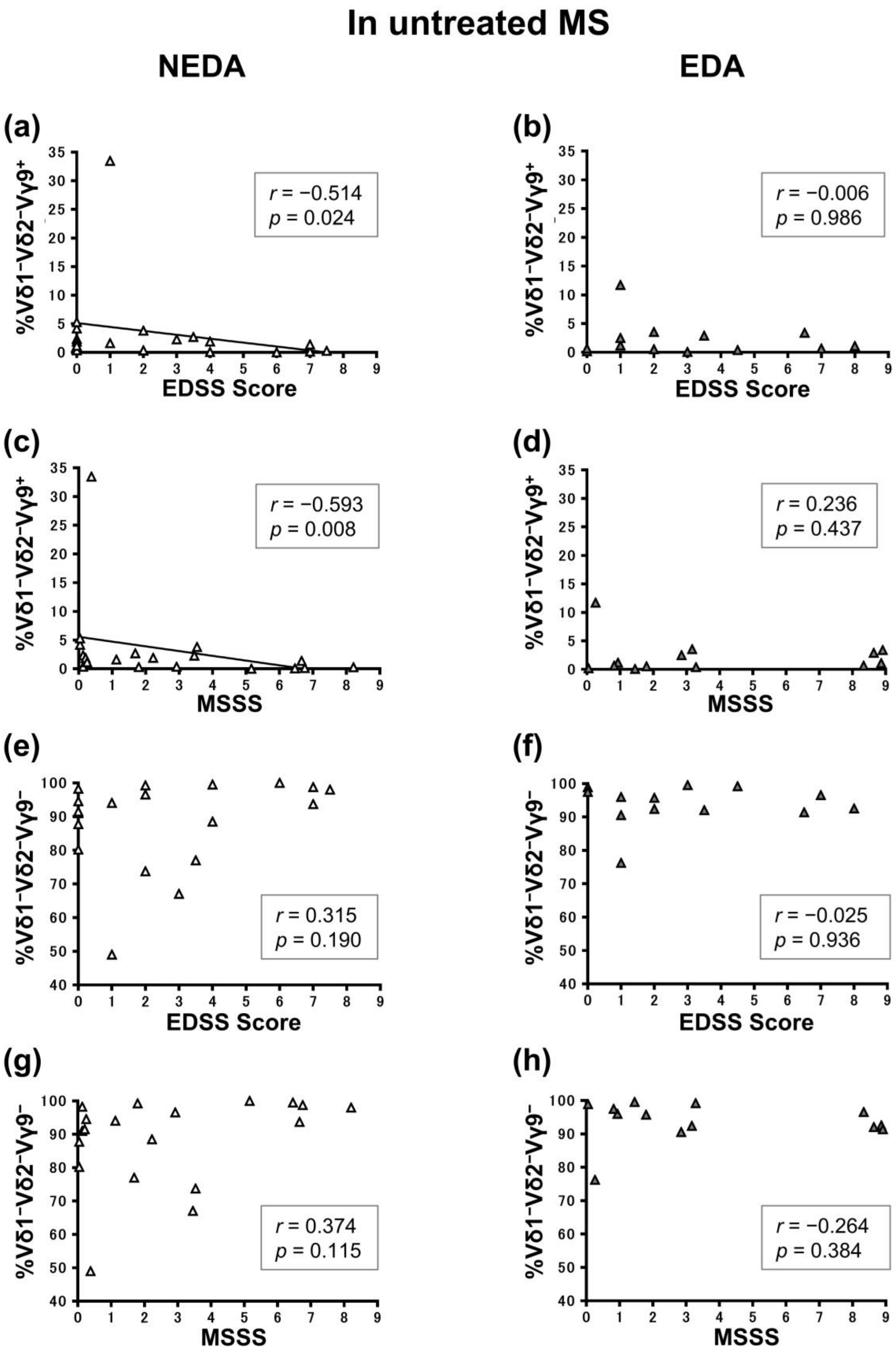

Figure S2 Correlations between the percentages of γδ T cell subsets and disability in untreated MS patients.

(**a-d**) Correlation between the percentage of $V\delta1^{-}V\delta2^{-}V\gamma9^{+}$ cells in γδ T cells and EDSS scores (**a, b**) or MSSS (**c, d**) at examination in untreated MS patients in the NEDA (**a, c**) and EDA (**e, f**) groups. (**e-h**) Correlation between the percentage of $V\delta1^{-}V\delta2^{-}V\gamma9^{-}$ cells in $V\delta1^{-}V\delta2^{-}$ γδ T cells and EDSS scores (**e, f**) or MSSS (**g, h**) at examination in untreated MS patients in the NEDA (**e, g**) and EDA (**f, h**) groups. Correlations were calculated using Spearman's rank correlation coefficient. Abbreviations: EDA = evidence of disease activity; EDSS = Expanded Disability Status Scale; MS = multiple sclerosis; MSSS = multiple sclerosis severity score; NEDA = no-evidence of disease activity.

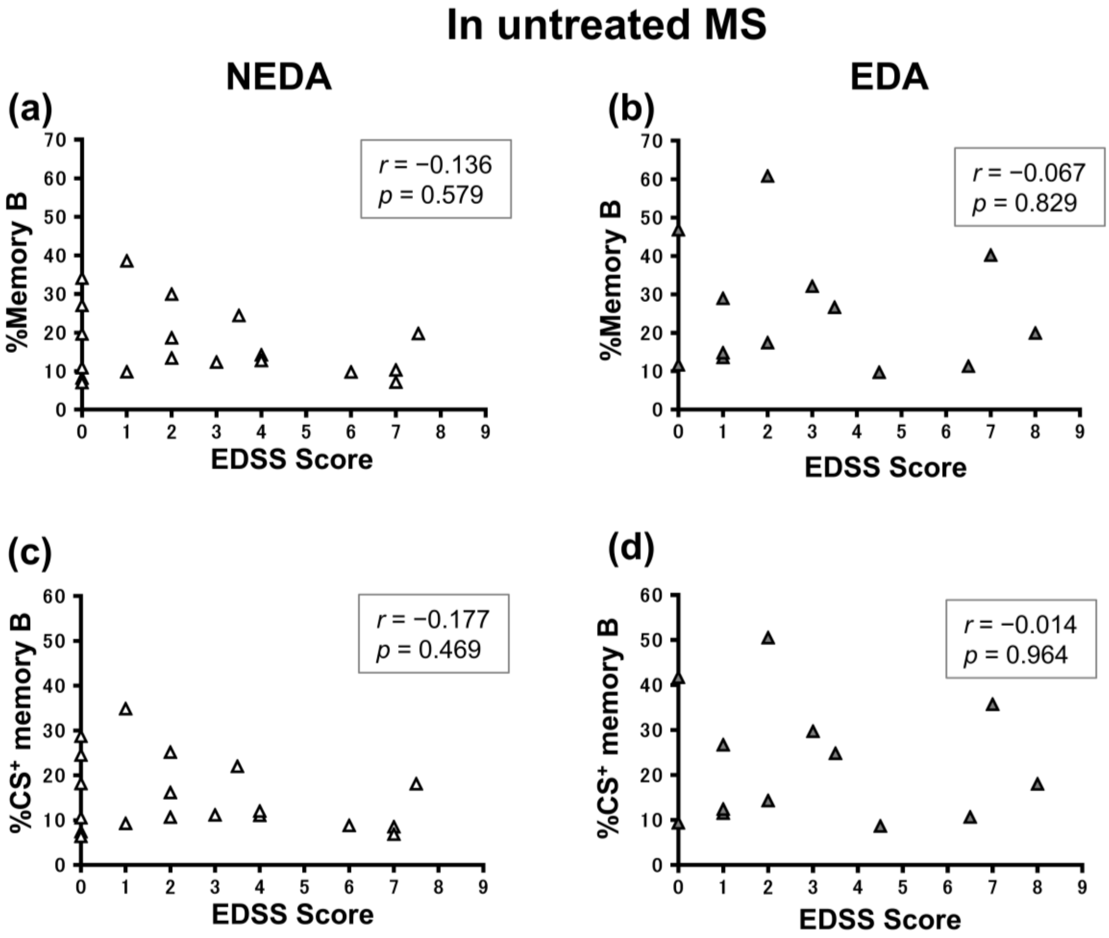

Figure S3 Correlations of memory B cell proportions with disability in untreated MS patients. (**a, b**) Correlation between the percentage of memory B cells and EDSS scores in untreated MS patients in the NEDA (**a**) and EDA (**b**) groups. (**c, d**) Correlation between the percentage of class-switched memory B cells and EDSS scores in untreated MS patients in the NEDA (**c**) and EDA (**d**) groups. Correlations were calculated using Spearman's rank correlation coefficient.

Abbreviations: CS^+ = class-switched; EDA = evidence of disease activity; MS = multiple sclerosis; NEDA = no-evidence of disease activity.
